# The Current Status of Immune Checkpoint Inhibitors in Neuro-Oncology: A Systematic Review

**DOI:** 10.3390/cancers12030586

**Published:** 2020-03-04

**Authors:** Cyrillo G. Brahm, Myra E. van Linde, Roelien H. Enting, Maaike Schuur, René H.J. Otten, Martijn W. Heymans, Henk M.W. Verheul, Annemiek M.E. Walenkamp

**Affiliations:** 1 Department of Medical Oncology, University of Groningen, University Medical Center Groningen, 9700 RB Groningen, The Netherlands; c.brahm@amsterdamumc.nl; 2 Department of Medical Oncology, Cancer Center Amsterdam, Amsterdam University Medical Centers, location VUmc, 1007 MB Amsterdam, The Netherlands; m.vanlinde@amsterdamumc.nl (M.E.v.L.); henk.verheul@radboudumc.nl (H.M.W.V.); 3 Department of Neurology, University of Groningen, University Medical Center Groningen, 9700 RB Groningen, The Netherlands; r.h.enting@umcg.nl; 4 Department of Neurology, Cancer Center Amsterdam, Amsterdam University Medical Centers, location VUmc, 1007 MB Amsterdam, The Netherlands; m.schuur@amsterdamumc.nl; 5 University Library, Vrije Universiteit Amsterdam, 1007 MB Amsterdam, The Netherlands; r.otten@vu.nl; 6 Department of Epidemiology and Biostatistics, Amsterdam University Medical Centers, location VUmc, 1007 MB Amsterdam, The Netherlands; mw.heymans@amsterdamumc.nl; 7 Department of Medical Oncology, Radboud University Medical Center, 6500 HB Nijmegen, The Netherlands

**Keywords:** Immune checkpoint inhibitors, glioblastoma, brain metastases, brain tumor, systematic review

## Abstract

The introduction of immune checkpoint inhibitors (ICI), as a novel treatment modality, has transformed the field of oncology with unprecedented successes. However, the efficacy of ICI for patients with glioblastoma or brain metastases (BMs) from any tumor type is under debate. Therefore, we systematically reviewed current literature on the use of ICI in patients with glioblastoma and BMs. Prospective and retrospective studies evaluating the efficacy and survival outcomes of ICI in patients with glioblastoma or BMs, and published between 2006 and November 2019, were considered. A total of 88 studies were identified (*n* = 8 in glioblastoma and *n* = 80 in BMs). In glioblastoma, median progression-free (PFS) and overall survival (OS) of all studies were 2.1 and 7.3 months, respectively. In patients with BMs, intracranial responses have been reported in studies with melanoma and non-small-cell lung cancer (NSCLC). The median intracranial and total PFS in these studies were 2.7 and 3.0 months, respectively. The median OS in all studies for patients with brain BMs was 8.0 months. To date, ICI demonstrate limited efficacy in patients with glioblastoma or BMs. Future research should focus on increasing the local and systemic immunological responses in these patients.

## 1. Introduction

Treating patients with primary brain tumors and brain metastases can be challenging. This is primarily due to the poor prognosis of these patients despite maximal treatment and the presence of the blood–brain barrier, posing an obstacle to overcome for most systemic treatments [1]. Glioblastoma is the most common and most aggressive primary brain tumor in adults, accounting for more than 50% of all gliomas. Currently, first-line standard treatment for patients with glioblastoma consists of maximal resection, followed by postoperative radiotherapy (RT) with concomitant and adjuvant temozolomide (TMZ) chemotherapy [2]. Since the addition of TMZ to postoperative treatment, two-year and five-year survival have improved to 27% and 10%, respectively [3]. Furthermore, the addition of tumor-treating fields, an anti-mitotic treatment modality, to TMZ maintenance therapy demonstrated a statistically significant improvement in progression-free and overall survival, compared to TMZ maintenance therapy alone (6.7 months vs. 4.0 months and 20.9 months vs. 16.0 months, respectively) [4]. However, recurrence is almost inevitable and therefore, the prognosis for these patients remains poor with a median survival of only 12–15 months [3]. At the time of recurrence, options are limited due to the distinct limitations in the use of surgery and re-irradiation, and the poor treatment response to chemotherapy and targeted therapy [5,6,7].

Brain metastases (BMs) occur in 8–10% of all cancer patients as an unfortunate complication of systemic dissemination [8,9]. The cumulative incidence of brain metastases is highest in melanoma (28%), followed by lung cancer (27%), renal cell cancer (11%), breast cancer (8%), and testicular cancer (8%) [10]. Similar to glioblastoma, most patients with brain metastases have a dismal prognosis of 12–15 months despite multidisciplinary treatment with surgery, irradiation and/or systemic treatment [11]. Therefore, there is an unmet need for more effective treatments for patients with glioblastoma or brain metastases.

Over the past few decades, significant progress has been made in the understanding of how cancer cells are able to evade the immune system through the expression of immune checkpoints that suppress T cell function and proliferation. Currently, the clinically most relevant immune checkpoints are the cytotoxic T lymphocyte antigen 4 (CTLA-4), the programmed death 1 receptor (PD-1) and its ligand (PD-L1) [12,13]. Interestingly, blockade of these immune checkpoints with antibodies, such as ipilimumab (anti-CTLA-4), nivolumab (anti-PD-1), and pembrolizumab (anti-PD-1), successfully demonstrated efficacy in various solid tumors, predominantly melanoma and non-small cell lung cancer (NSCLC), and prolonged the survival of patients with extracranial disease [14,15,16].

The introduction of immune checkpoint inhibitors (ICI), as an unprecedented treatment modality, has consequences for clinical decision making of neuro-oncologists and treatment recommendations of Neuro-Oncology tumor boards. Furthermore, these treatment recommendations and decisions may differ per tumor type. Therefore, we systematically reviewed and summarized current literature on the use of checkpoint inhibitors in patients with glioblastoma and brain metastases to support neuro-oncologists and neuro-oncology tumor boards in their clinical decision making and treatment recommendations.

## 2. Methods

### 2.1. Literature Search

The systematic review followed the guidelines of the Preferred Reporting Items for Systematic Reviews and Meta-Analysis (PRISMA)-statement (http://www.prisma-statement.org). PubMed, EMBASE.com and the Cochrane Library (via Wiley) were searched for potentially eligible publications from inception (by C.B. and R.O.) up to 11 November 2019. The following keywords (including synonyms and closely related words) were used as index terms or free-text words: ‘glioblastoma OR gbm’ or ‘brain metastases OR central nervous system metastases’ and ‘immunotherapy OR immune checkpoint inhibitor’. A full overview of the complete search strategies can be found in supplementary Table S1. Subsequently, the titles and abstracts found by the database searches were exported to a reference manager database to remove all duplicate articles.

### 2.2. Study Selection

Eligible studies included (i) prospective or retrospective studies in patients with glioblastoma or brain metastases, (ii) reporting on the efficacy and survival outcomes after treatment with immune checkpoint inhibitors and (iii) were published in English between January 2006 and the end of November 2019. Case reports, scientific abstracts or studies with <10 patients were excluded. In the first selection phase, two authors (C.B. and M.L.) independently screened and reviewed the titles and abstracts of all identified articles.

### 2.3. Data Extraction and Statistical Analysis

The two reviewers (C.B. and M.L.) extracted the following data from each article: author (year), study design, tumor type, therapeutic agent and dose, type of radiation therapy, number of participants, objective response rate (ORR), local brain metastasis control rate at 6 and 12 months, distant brain metastasis control rate at 6 and 12 months, progression-free survival (PFS), and overall survival (OS). If criteria other than RANO or RECIST were used, these were taken into consideration for calculating the ORR. In case ORR was not reported in an article, it was calculated from raw proportions of events (complete response (CR), partial response (PR), or stable disease (SD) divided by the total number of evaluable patients. Descriptive analyses were performed to assess outcomes and demographic characteristics. Freeman-Tukey double arcsine transformation of proportions was applied before pooling these values and results were back transformed. A random effects model was used for all pooled proportions. Forest plots were generated to show the prevalence of each study and the overall pooled prevalence and I^2^ statistics were calculated. R software version 3.6.1 (package meta) was used to perform meta-analyses.

## 3. Results

### 3.1. Literature Search Results

**Figure 1:**
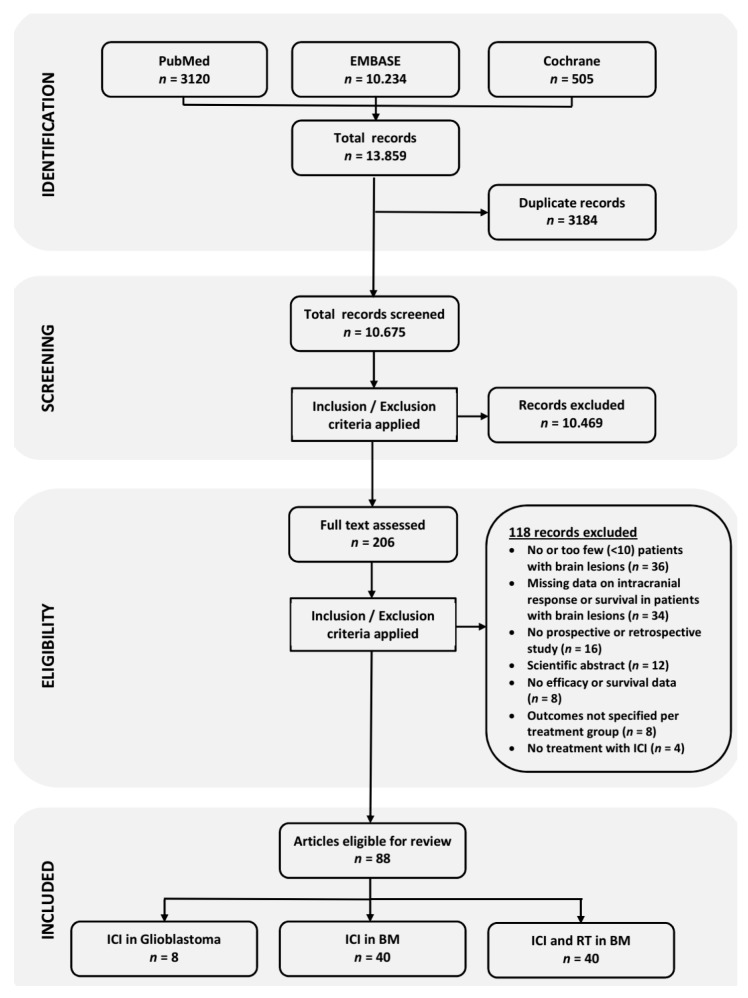
Flow diagram illustrating the literature search and study selection.

A flow diagram of the literature search, review and selection process is illustrated in Figure 1. In total, the literature search yielded 10,675 individual records, after removal of duplicate records. Screening of the titles and abstracts of these records resulted in 206 records eligible for full-text assessment. Finally, 118 records were excluded after reading the full text, resulting in 88 eligible publications for this review.

### 3.2. Overview of the Studies

For glioblastoma, a total of 8 studies were included in this systematic review, including two phase I trials [17,18], two phase II trials [19,20] and four retrospective analyses [21,22,23,24] (Table 1). In two studies, pembrolizumab was administered in patients with recurrent glioblastoma every three weeks (Q3W) in varying doses [19,22]. Three studies used nivolumab, administered most frequently in a dose of 3 mg/kg every two weeks (Q2W) [17,23,24]. Ipilimumab was only used in combination with bevacizumab [21] or nivolumab [17]. Lastly, one study used the PD-L1 inhibitor atezolizumab, administered 1200 mg Q3W [18].

For brain metastases, 40 studies assessed the efficacy of ICI without RT [25,26,27,28,29,30,31,32,33,34,35,36,37,38,39,40,41,42,43,44,45,46,47,48,49,50,51,52,53,54,55,56,57,58,59,60,61,62,63,64] and 40 studies explored the combination of ICI and RT (Table 2; Tables S2–S4). The 41 studies with ICI in BMs include three phase III trials, eleven phase II trials, ten Extended Access Program (EAP) studies and 17 retrospective analyses. Aside from one phase I trial, all of the other studies with ICI and RT in BMs were retrospective analyses with a highly heterogeneous study design. Therefore, the outcomes of studies with ICI and RT are reported separately in the supplementary data (Tables S3 and S4). The most common tumor types in all studies of ICI with or without RT were melanoma and NSCLC.

### 3.3. Immune Checkpoint Inhibitors in Glioblastoma

The efficacy and survival outcomes of all studies are summarized in Table 1. The median survival of the patients with glioblastoma treated with ICI in all studies is 7.3 months. Furthermore, the median PFS reported in these studies was 2.1 months (Table 3).

Overall, objective responses to ICI in recurrent glioblastoma were seen in three studies [17,18,21]. In the retrospective analysis of Carter et al., an ORR of 31% was seen in patients with recurrent glioblastoma, who were treated with ipilimumab, 3 mg/kg Q3W, combined with bevacizumab [21]. In the phase I trial reported by Omuro et al., in 2018, objective responses were seen in two of the three treatment arms. The ORR in the treatment arms with nivolumab monotherapy (3 mg/kg Q2W) and nivolumab (3 mg/kg Q2W) combined with ipilimumab (1 mg/kg) were 11% and 10%, respectively [17]. Lastly, the phase I study of Lukas et al., with atezolizumab (1200 mg Q3W) in 16 patients with glioblastoma showed an ORR of 6.0% [18]. Three patients with IDH1-mutant tumors had better PFS (5.5 months vs. 1.2 months) and a trend towards a longer OS (16.0 months vs. 2.7 months) than patients with IDH1-wild-type tumors. Interestingly, neoadjuvant PD-1 blockade with pembrolizumab in patients with recurrent, surgically resectable glioblastoma demonstrated a significant improvement in OS compared to adjuvant PD-1 blockade alone. Furthermore, neoadjuvant PD-1 blockade was associated with upregulation of T cell and interferon-γ-related gene expression, but downregulation of genes related to the cell-cycle in the tumor [19]. Similar intratumoral and systemic immune changes were found in a single-arm, phase II trial with neoadjuvant nivolumab in surgically resectable, newly diagnosed or recurrent glioblastoma [20]. Currently, three important studies with nivolumab in newly-diagnosed and recurrent glioblastoma are awaiting final publication, but have reported preliminary, disappointing primary outcomes (Table 4). First, in the randomized, open-label, phase III CheckMate-143 trial (NCT02017717), nivolumab monotherapy did not significantly improve overall survival in patients with recurrent glioblastoma, compared to treatment with bevacizumab [65]. Furthermore, the combination of nivolumab with radiotherapy in the CheckMate-498 trial (NCT02617589) also failed to significantly prolong the overall survival of patients with newly diagnosed O^6^-methylguanine-DNA methyltransferase (*MGMT*)-unmethylated glioblastoma, compared to combined treatment with temozolomide and radiotherapy. Lastly, the addition of nivolumab to the first-line treatment with temozolomide and radiotherapy in newly diagnosed *MGMT*-methylated glioblastoma patients (CheckMate-548; NCT02667587), failed to meet one of its primary endpoints, i.e., PFS, and is currently awaiting the overall survival data.

### 3.4. Outcomes of Immune Checkpoint Inhibitors in Brain Metastases

An overview of the efficacy and survival outcomes is provided in Table 2 and Supplementary Table S2 for all studies with checkpoint inhibitors in brain metastases, and Table S3 for the studies that combined checkpoint inhibitors with radiotherapy. The median intracranial PFS for patients with brain metastases treated with ICI in these studies is 2.7 months, compared to an overall PFS of 3.0 months. The median survival of these patients reported in the studies was 8.0 months (Table 3).

**Figure 2:**
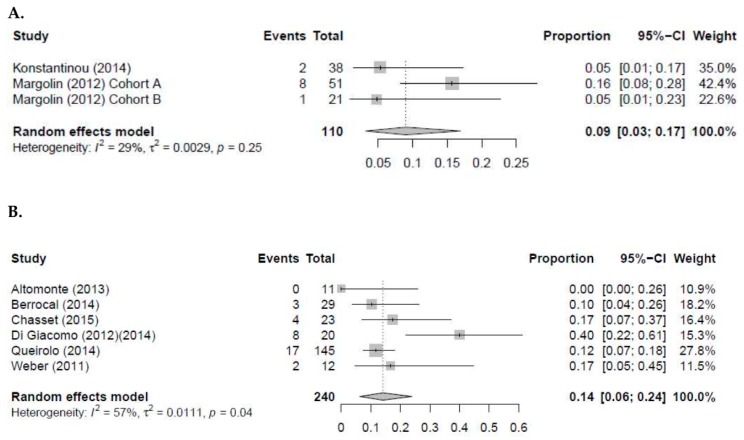
Pooled analysis for intracranial ORR (**A**) and overall ORR (**B**) of ipilimumab in patients with melanoma brain metastases.

In patients with melanoma BMs, 13 studies explored the efficacy of ipilimumab monotherapy. The pooled intracranial objective response rate (iORR) and ORR for ipilimumab are 9.0% (95% CI 3.0–17; I^2^ = 29%) and 14.0% (95% CI 6.0–24.0 I^2^ = 57%), respectively (Figure 2) [25,26,28,30,31,39,41,44,46,48,49,50,55]. Furthermore, the median survival in these studies ranged from 3.3 to 14.0 months and the intracranial PFS ranged from 1.2 to 3.0 months. For pembrolizumab monotherapy, four studies conducted in patients with melanoma BMs reported an intracranial ORR 22.0–40.0% (Figure S1) [36,37,38,51]. The median PFS and OS of these patients were 2.0 to 5.2 months and 17.0–20.4 months, respectively. Long et al., and Larkin et al., explored the efficacy of nivolumab monotherapy in melanoma BMs [57]. The median survival and PFS for patients with BMs in these studies ranged from 5.1 to 18.5 months and 2.3 to 2.5 months, respectively. Furthermore, the reported intracranial ORR was 6.0–20.0%. Lastly, three studies explored the efficacy of ipilimumab combined with nivolumab [40,42,47]. In two of the three studies, the median OS and PFS were not reached. However, Parakh et al., reported a median OS of 17.4 months (95% CI 7.1–N.R.) and a PFS of 2.9 months (95% CI 0.6–7.1) [42]. Interestingly, the intracranial ORR in these three studies were 18.0–55.0% (Figure S2).

In NSCLC brain metastases, eight studies reported efficacy and survival outcomes of treatment with ICI. In six studies with nivolumab monotherapy, the patients had a median survival of 3.1 to 9.0 months [29,32,34,35,56,62]. Furthermore, median PFS ranged from 1.6 to 3.0 months. The pooled ORR in these studies was 14% (95% CI 8.0–23.0; I^2^ = 29%) (Figure S3). Subsequently, Goldberg et al., reported an intracranial ORR of 33.0% (95% CI 14.0–59.0) in patients with NSCLC BMs treated with pembrolizumab [36]. The median survival of these patients was 7.7 months (95% CI 3.5–N.R.). Lastly, Spigel et al., explored the efficacy of atezoluzimab in a cohort of patients with NSCLC and treated BMs [60]. The ORR in this cohort was 23.0% (95% CI 5.0–54.0) and patients had a PFS and OS of 2.5 months and 6.8 months (95% CI 3.2–19.5), respectively.

In other solid malignancies, only four studies reported efficacy and/or survival outcomes of patients with brain metastases, who were treated with ICI. First, in the phase II trial of Gadgeel et al., patients with small cell lung cancer (SCLC) were treated with pembrolizumab, 200 mg Q3W [54]. The median survival of these patients was 9.6 months (95% CI 7.0–12.0) and patients had a median PFS of 1.4 months (95% CI 1.2–2.8). Second, Flippot et al., and De Giorgi et al., reported an intracranial ORR of 11.8% and an ORR of 18.8%, respectively, in patients with renal cell cancer (RCC), who were treated with nivolumab, 3 mg/kg Q2W [33,52]. The median survival was not reached or available in these studies. However, Flippot et al., reported a median intracranial PFS 2.7 months (95% CI 2.3–4.6), compared to 4.8 months (95% CI 3.0–8.0) for patients with RCC BMs, who were treated with nivolumab and local therapy. The median PFS of the patients in the study of De Giorgi et al., was 4.4 months (95% CI 3.7–6.2). Lastly, the study of Sternberg et al., observed no objective responses in patients with BMs from urothelial and non-urothelial urinary tract carcinoma (UTC), treated with atezolizumab 1200 mg Q3W [45]. The median PFS and OS of the patients in this study were 2.0 months (95% CI 1.5–2.3) and 3.7 months (95% CI 1.5–7.0), respectively.

## 4. Discussion

In this systematic review, we examined the efficacy and survival outcomes of checkpoint inhibitors in patients with glioblastoma and brain metastases.

For glioblastoma, we found that the vast majority of the studies with checkpoint inhibitors in recurrent glioblastoma showed minimal clinical activity. Furthermore, although currently awaiting final publication, preliminary results of the three landmark CheckMate studies with nivolumab have failed to meet their primary endpoint [65]. Based on these results, the use of immune checkpoint inhibitors is not applicable for patients with glioblastoma. Aside from the blood–brain barrier, which affects the drug delivery in brain tumors, it is suggested that several factors play an important role in the limited efficacy of checkpoint inhibitors in glioblastoma, compared to brain metastases of solid tumors. First, glioblastoma generally exhibits a relatively low mutational load compared to other solid tumors (i.e., melanoma and NSCLC), with an exemption of the infrequent cases of glioblastoma in which there is a defective mismatch repair system, resulting in a higher mutational load [66,67]. Furthermore, in contrast to melanoma and NSCLC brain metastases and their primary tumors, the gene expression signature of glioblastoma induces a highly immunosuppressive microenvironment, including a relatively low neoantigen burden and low number of tumor-infiltrating lymphocytes (TILs) [68,69]. Therefore, to overcome these difficulties, a multimodal, molecular approach may be necessary to increase the immune microenvironment and anti-tumor response in a selected subgroup of patients with glioblastoma.

Intracranial responses have been reported in studies with melanoma and NSCLC brain metastases. While multiple studies in melanoma brain metastases report intracranial objective responses with anti-PD-1 and/or anti-CTLA-4 immunotherapy, the evidence in NSCLSC BMs is limited to anti-PD-1 immunotherapy (i.e., predominantly nivolumab). Despite the encouraging evidence to presume that ICI can demonstrate intracranial objective responses in patients with brain metastases, the survival outcomes for these patients remain poor. Patients with brain metastases in the reviewed literature had a median intracranial and total PFS of 2.7 and 3.0 months, respectively. Furthermore, the reported median OS in all the reviewed studies was only 8.0 months. Several factors might play a role for the limited number of responses and poor PFS reported in patients with melanoma and NSCLC brain metastases. First, as with glioblastoma, the number of TILs in the microenvironment of brain metastases is highly heterogeneous and differs between patients [70]. Second, the density of these TILs seems to be significantly correlated with the amount of peritumoral brain edema and survival outcome in patients with brain metastases [71]. Lastly, it is hypothesized that the use of corticosteroids, which are frequently administered in patients with brain metastases and glioblastoma for the management of cerebral edema, may restrain a tumor-specific immune response to checkpoint inhibition by impairing T lymphocyte activation [41,72]. In particular, baseline use of steroids prior to the initiation of ICI seems to be correlated with a decreased ORR, PFS and OS, while the use of corticosteroid after initiation of ICI is not [73,74].

Currently, only a small subset of patients with glioblastoma or BMs with microsatellite instable or mismatch repair deficient tumors, resulting in a higher tumor mutational burden, may benefit from ICI. Therefore, new treatment strategies are necessary to increase the response to ICI in patients with glioblastoma or BMs. First, the use of ICI in combination or in sequence with radiotherapy has mostly been explored in retrospective studies. Unfortunately, in the absence of randomized, prospective data, it is difficult to draw any conclusion about the optimal timing and sequencing of radiotherapy with ICI from these studies. Second, a potential way to overcome the highly immunosuppressive tumor microenvironment and low number of TILs is by inhibiting histone deacetylases (HDAC). Inhibition of HDACs lead to increased histone acetylation, resulting in increased gene expression [75]. Recently, both the HDAC inhibitor mocetinostat and inhibition of HDAC6 independently demonstrated a synergistic effect in combination with ICI, resulting in increased anti-tumor activity in NSCLC and ovarian cancer cell lines by increasing tumor antigen presentation and decreasing immune suppressive cell types [76,77]. Furthermore, in a phase I study, an adenovirus vector encoding the IL-12 gene was injected during surgery in the resection cavity walls of patients with recurrent high-grade glioma, followed by post-operative treatment with the oral activator for human IL-12, veledimex. In the tissue of five patients that received a re-resection, increased tumor-infiltrating lymphocytes producing interferon-γ and PD-1 were seen, supporting the hypothesis of an immunological antitumor effect of human IL-12 [78]. Collectively, these data suggest that a multimodal approach is necessary to increase the activation of the immune system in the tumor microenvironment and anti-tumor response to ICI in patients with glioblastoma or BMs.

A few important limitations should be considered in the interpretation of the results reported in this systematic review. First, given the limited number of available randomized controlled trials and the retrospective nature of several included studies, the results in these studies are subjected to a certain degree of selection bias and therefore the real-world data may be worse. Subsequently, a considerable heterogeneity exists in the reported data of the studies, most likely due to the differences in study design, number of patients, study treatment and disease evaluation. A random effects models was used for our pooled analysis of ORR to minimize the bias in these data. Second, not every study included in this systematic review had complete data available for all the outcomes of interest. Furthermore, data on microsatellite stability, mismatch repair deficiency and tumor mutational burden were lacking. Therefore, unfortunately, no conclusions can be drawn about the correlation between the genetic profile and response to ICI. Lastly, most of the included studies predominantly focused on the use of ICI in melanoma BMs, compared to a select number of studies in NSCLC BMs and minimal data available in BMs of other solid tumors for which ICI are FDA-approved. Therefore, the outcomes of the studies with ICI in NSCLC BMs should be interpreted with caution and primarily in the setting of melanoma and NSCLC.

## 5. Conclusions

In conclusion, immune checkpoint inhibition, in its current state, demonstrates limited efficacy in glioblastoma and has failed to improve the survival of these patients. Therefore, for the future of immunotherapy in glioblastoma, research should focus on a multi-modal approach to activate local and systemic tumor-specific immune responses in glioblastoma. In patients with melanoma and NSCLC brain metastases, intracranial objective responses are seen with checkpoint inhibitors. However, due to the relatively poor overall survival, intracranial and total PFS with checkpoint inhibitors, local and systemic personalized treatment recommendations should be discussed in a multidisciplinary neuro-oncology tumor board. To move the field of checkpoint inhibition in brain metastases forward, we suggest more and larger prospective randomized controlled trials for patients with brain metastases. This must result in comprehensive evidence of the therapeutic potential of FDA-approved immune checkpoint inhibitors in brain metastases and subsequent determination of whether checkpoint inhibition improve quality of life and overall survival for these patients.

## Figures and Tables

**Table 1:** Primary outcomes of clinical studies with immune checkpoint inhibitors in patients with glioblastoma.

Author	Study Design	Tumor Type	Setting	Agent	No. of Patients	ORR	PFS	OS
						*%*	Months	Months
Carter (2016) [21]	Retrospective	Glioblastoma	Recurrent	IPI + BEV	16	31.0	N.A.	N.A.
Blumenthal (2016) [22]	Retrospective	Glioma	Recurrent	PEMBRO	17 (10 GBM)	0.0	N.A.	2.6 [range 0.4–11.6]
Chamberlain (2017) [23]	Retrospective	Glioblastoma	Recurrent	NIVO	16	0.0	2.0 (95% CI 1.3–2.7)	3.5 (95% CI 2.8–4.2)
Omuro (2018) Cohort A [17]	I	Glioblastoma	Recurrent	NIVO	10	11.0	1.9 (95% CI 1.3–4.6)	10.4 (95% CI 4.1–22.8)
Omuro (2018) Cohort B	I	Glioblastoma	Recurrent	NIVO + IPI	10	0.0	1.5 (95% CI 0.5–2.8)	9.2 (95% CI 3.9–12.7)
Omuro (2018) Cohort C	I	Glioblastoma	Recurrent	NIVO + IPI	20	10.0	2.1 (95% CI 1.4–2.8)	7.3 (95% CI 4.7–12.9)
Mantica (2018) [24]	Retrospective	Glioblastoma	Recurrent	NIVO (+ BEV)	37	0.0	4.6 [range 0.5–15.0]	6.5 [range 0.8–19.5]
Lukas (2018) [18]	I	Glioblastoma	Recurrent	ATEZO	16	6.0	1.2 [range 0.7–10.7]	4.2 [range 1.2–18.8+]
Cloughesy (2019) Cohort A [19]	II	Glioblastoma	Recurrent	PEMBRO	16	N.A.	3.3	13.7
Cloughesy (2019) Cohort B [19]	II	Glioblastoma	Recurrent	PEMBRO	16	N.A.	2.4	7.5
Schalper (2019) [20]	II	Glioblastoma	Newly diagnosed and Recurrent	NIVO	29	N.A.	4.1 (95% CI 2.8–5.5)	7.3 (95% CI 5.4–7.9)

ATEZO Atezolizumab; BEV Bevacizumab; ICI Immune checkpoint inhibitor; IPI Ipilimumab; N.A. Not available; NIVO Nivolumab; PEMBRO Pembrolizumab.

**Table 2:** Primary outcomes of clinical studies with immune checkpoint inhibitors in patients with brain metastases.

Author	Study Design	Tumor Type	Agent	No. of Patients	ORR	PFS	OS
					*%*	Months	Months
Altomonte (2013) [25]	EAP (Retrospective)	Melanoma	IPI	11	0	3.0 (95% CI 2.4–3.6)	4.0 (95% CI 2.4–5.6)
Berrocal (2014) [26]	EAP (Retrospective)	Melanoma	IPI	29	10.8	N.A.	3.9 (95% CI 1.1–6.8)
Chasset (2015) [28]	EAP (Retrospective)	Melanoma	IPI	23	17	N.A.	7.0 (95% CI 4.0–12.0)
Di Giacomo (2012)(2014) [30,31]	II	Melanoma	IPI + Fotemustine	20	40.0	3.0 † (95% CI 2.9–3.1)	12.7 (95% CI 2.7–22.7)
Goldberg (2016) [36]	II	Melanoma	PEMBRO	18	22.0 * (95% CI 7.0–48.0)	N.A.	N.R.
González-Cao (2017) [37]	EAP (Retrospective)	Melanoma	PEMBRO	10	40.0 *	N.A.	N.A.
Kluger (2019) [38]	II	Melanoma	PEMBRO	23	26.0 * (95% CI 10.0–48.0)	2.0 (95% CI, 2.0–N.R.)	17.0 (95% CI 10.0–N.R.)
Konstantinou (2014) [39]	EAP (Retrospective)	Melanoma	IPI	38	5.3 *	N.A.	3.3
Long (2018) Cohort A [40]	II	Melanoma	NIVO + IPI	35	46.0 * (95% CI 29.0–63.0)	N.R. † (95% CI 2.9–N.R)	N.R. (95% CI 8.5–N.R.)
Long (2018) Cohort B [40]	II	Melanoma	NIVO	25	20.0 * (95% CI 7.0–41.0)	2.5 † (95% CI 1.7–2.8)	18.5 (95% CI 6.9–N.R.)
Long (2018) Cohort C [40]	II	Melanoma	NIVO	16	6.0 * (95% CI 0.0–30.0)	2.3 † (95% CI 1.4–4.3)	5.1 (95% CI 1.8–N.R.)
Margolin (2012) Cohort A [41]	II	Melanoma	IPI	51	16.0 * (95% CI 7.0–29.0)	1.5 † (95% CI 1.2–2.5)	7.0 (95% CI 4.1–10.8)
Margolin (2012) Cohort B [41]	II	Melanoma	IPI	21	5.0 * (95% CI 0.1–24.0)	1.2 † (95% CI 1.2–1.3)	3.7 (95% CI 1.6–7.3)
Parakh (2017) [43]	Retrospective	Melanoma	NIVO or PEMBRO	66	21.0 *	5.3 † (95% CI 3.3–8.2)	9.9 (95% CI 6.9–17.7)
Parakh (2019) [42]	Retrospective	Melanoma	NIVO + IPI	11	18.0 *	2.9 (95% CI 0.6–7.1)	17.4 (95% CI 7.1–N.R.)
Queirolo (2014) [44]	EAP (Retrospective)	Melanoma	IPI	145	12.0	3.1 (95% CI 2.7–3.5)	4.3 (95% CI 3.4–5.2)
Tawbi (2018) [47]	II	Melanoma	NIVO + IPI	94	55.0 * (95% CI 45–66)	N.R.	N.R.
Weber (2011) [46]	II	Melanoma	IPI (+ Budesonide)	12	16.7	N.A.	14.0
Bjørnhart (2019) [27]	Retrospective	NSCLC	NIVO or PEMBRO	21	4.8 *	4.2 (95%CI 2.5–5.9)	8.2 (95% CI 1.0–15.5)
Crinò (2019) [29]	EAP	NSCLC	NIVO	409	17.0	3.0 (95% CI 2.7–3.3)	8.6 (95% CI 6.4–10.8)
Dumenil (2018) [32]	Retrospective	NSCLC	NIVO	10	0	N.A.	3.1
Garde-Noguera (2018) [34]	Retrospective	NSCLC	NIVO	38	17.2	1.6	3.1
Gauvain (2018) [35]	Retrospective	NSCLC	NIVO	30	9.0 * (95% CI 3.0–23.0)	3.9 † (95% CI 2.8–11.1)	N.R.
Goldberg (2016) [36]	II	NSCLC	PEMBRO	18	33.0 * (95% CI 14.0–59.0)	N.A.	7.7 (95% CI 3.5–N.R.)
Spigel (2018) Cohort 3 [60]	II	NSCLC	ATEZO	13	23.0 (95% CI 5.0–54.0)	2.5	6.8 (95% CI 3.2–19.5)
Flippot (2019) Cohort A [33]	II	RCC	NIVO	39	11.8 * (95% CI 3.3–27.5)	2.7 † (95% CI 2.3–4.6)	N.A.
Flippot (2019) Cohort B [33]	II	RCC	NIVO + Local Tx	34	N.A.	4.8 † (95% CI 3.0–8.0)	N.A.
Sternberg (2019) [45]	III	UTC	ATEZO	14	0 (95% CI 0–23.0)	2.0 (95% CI 1.5–2.3)	3.7 (95% CI 1.5–7.0)

* Intracranial ORR; † Intracranial PFS. EAP Expanded access program; ICI Immune checkpoint inhibitor; IPI Ipilimumab; N.A. Not available; NIVO Nivolumab; N.R. Not reached; PEMBRO Pembrolizumab; UTC Urinary Tract Cancer.

**Table 3:** Median survival outcomes of immune checkpoint inhibitors in patients with glioblastoma or brain metastases.

Variable	Glioblastoma	Brain Metastases							
		Melanoma				NSCLC			All
		IPI	NIVO	PEMBRO	IPI + NIVO	PEMBRO	NIVO	ATEZO	All
Median Intracranial PFS	2.1 mo	1.2–3.0 mo	2.3–2.5 mo	N.A.	N.A.	N.A.	3.9 mo	N.A.	2.7 mo
Median PFS	-	3.0–3.1 mo	N.A.	2.0–5.2 mo	2.9 mo	N.A.	1.6–3.0 mo	2.5 mo	3.0 mo
Median OS	7.3 mo	3.3–14.0 mo	5.1–18.5 mo	17.0–20.4 mo	17.4 mo	7.7 mo	2.8–9.0 mo	6.8 mo	8.0 mo

ATEZO: Atezolizumab; PFS: Progression-free survival; OS: Overall survival; IPI: Ipilimumab; NIVO: Nivolumab; PEMBRO: Pembrolizumab. N.A.: Not available; mo: Months.

**Table 4:** Important studies with immune checkpoint inhibitors in newly-diagnosed and recurrent glioblastoma awaiting publication.

NCT Number	Official Trial Name	Phase	Primary Endpoint	Endpoint Status
NCT02017717	A Study of the Effectiveness and Safety of Nivolumab Compared to Bevacizumab and of Nivolumab With or Without Ipilimumab in Glioblastoma Patients (CheckMate-143)	III	Overall survival	Endpoint not met
NCT02617589	An Investigational Immuno-therapy Study of Nivolumab Compared to Temozolomide, Each Given With Radiation Therapy, for Newly-diagnosed Patients With Glioblastoma (GBM, a Malignant Brain Cancer) (CheckMate-498)	III	Overall survival	Endpoint not met
NCT02667587	An Investigational Immuno-therapy Study of Temozolomide Plus Radiation Therapy With Nivolumab or Placebo, for Newly Diagnosed Patients With Glioblastoma (GBM, a Malignant Brain Cancer) (CheckMate-548)	III	Progression-free survival Overall survival	Endpoint not met Endpoint in progress

## Supplementary Materials

The following are available online at https://www.mdpi.com/2072-6694/12/3/586/s1, Figure S1: Pooled analysis for intracranial ORR of pembrolizumab in patients with melanoma brain metastases, Figure S2. Pooled analysis for intracranial ORR of nivolumab combined with ipilimumab in patients with melanoma brain metastases, Figure S3. Pooled analysis for ORR of nivolumab in patients with NSCLC brain metastases, Table S1. Full overview of the complete search strategies, Table S2. Studies with immune checkpoint inhibitors in patients with brain metastases that only reported overall survival data, Table S3. Primary outcomes of clinical studies with immune checkpoint inhibitors and radiotherapy in patients with brain metastases, Table S4. Clinical studies with immune checkpoint inhibitors and radiotherapy in patients with brain metastases that only reported overall survival data.
