# Supplementary material for: The Current Status of Immune Checkpoint Inhibitors in Neuro-Oncology: A Systematic Review
Source: Cancers (Basel). 2020 Mar 4;12(3):586. doi: 10.3390/cancers12030586 (PMC7139638; doi:10.3390/cancers12030586)
Supplement: Supplementary file 1 [file cancers-12-00586-s001.pdf]

Supplementary Materials: The Current Status of Immune Checkpoint Inhibitors in Neuro-Oncology: A Systematic Review

Cyrillo G. Brahm, Myra E. van Linde, Roelien H. Enting, Maaike Schuur, René H.J. Otten, Martijn W. Heymans, Henk M.W. Verheul and Annemiek M.E. Walenkamp

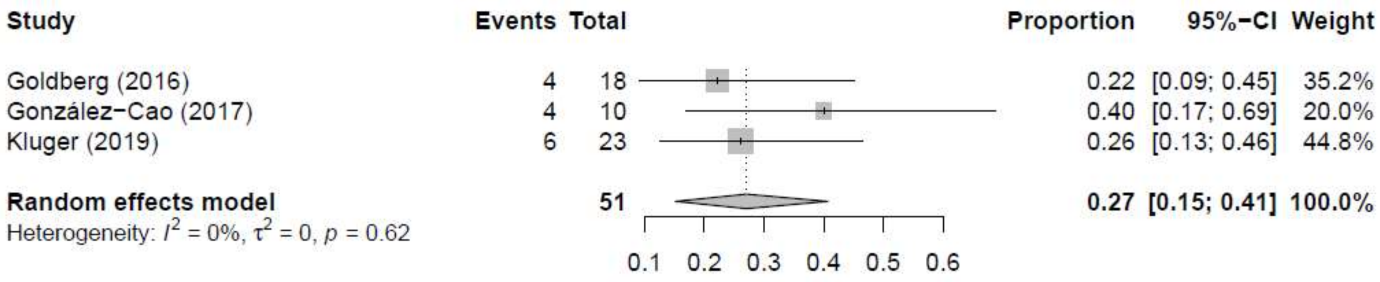

Figure S1. Pooled analysis for intracranial ORR of pembrolizumab in patients with melanoma brain metastases.

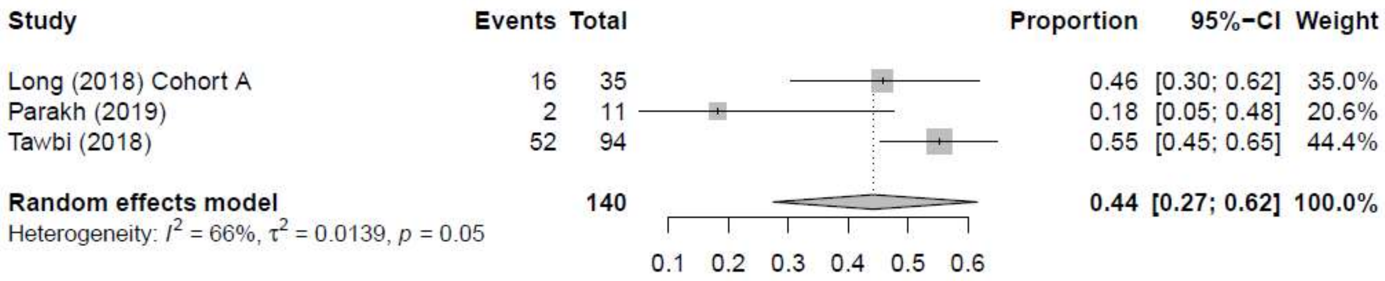

Figure S2. Pooled analysis for intracranial ORR of nivolumab combined with ipilimumab in patients with melanoma brain metastases.

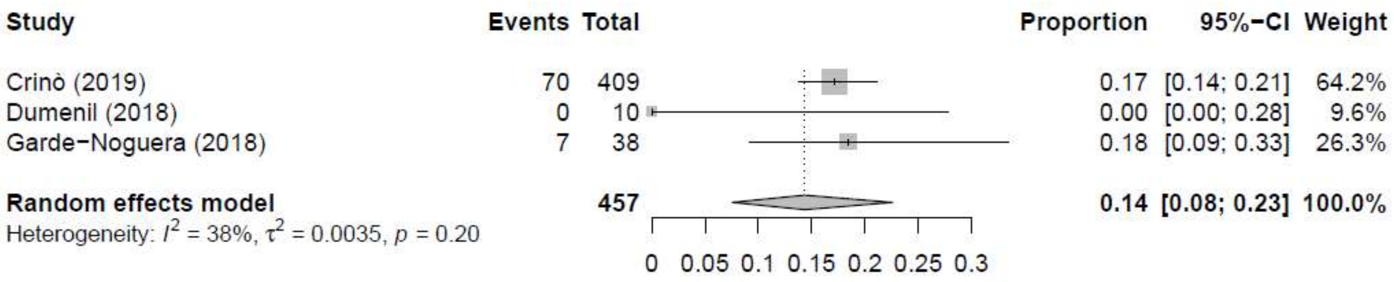

Figure S3. Pooled analysis for ORR of nivolumab in patients with NSCLC brain metastases.

Table S1. Full overview of the complete search strategies.

Search strategy in PubMed (2019 November 11th)

#	Query	Results
**#15**	#13 OR #14	**3120**
**#14**	#8 AND #12	**1542**
**#13**	#1 AND #12	**1704**
**#12**	#9 OR #10 OR #11	**327442**
**#11**	"CTLA-4 Antigen"[Mesh] OR "Programmed Cell Death 1 Receptor"[Mesh] OR "B7-H1 Antigen"[Mesh] OR “CTLA 4 Antigen”[tiab] OR “CTLA 4 Antigens”[tiab] OR “CTLA4 Antigen”[tiab] OR “CTLA4 Antigens”[tiab] OR “Cytotoxic T-Lymphocyte-Associated Antigen 4”[tiab] OR “Cytotoxic T-Lymphocyte Antigen 4”[tiab] OR “CD152 Antigen”[tiab] OR “CD152 Antigens”[tiab] OR “B7 H1 Antigen”[tiab] OR “B7 H1 Antigens”[tiab] OR “Programmed Cell Death 1 Ligand 1”[tiab] OR “B7-H1 Immune Costimulatory Protein”[tiab] OR “B7-H1 Immune Costimulatory Proteins”[tiab] OR “PD-L1 Costimulatory Protein”[tiab] OR “PD-L1 Costimulatory Proteins”[tiab] OR “Programmed Cell Death 1 Ligand 1 Protein”[tiab] OR “CD274 Antigen”[tiab] OR “CD274 Antigens”[tiab] OR “B7H1 Immune Costimulatory Protein”[tiab] OR “B7H1 Immune Costimulatory Proteins”[tiab] OR “PD-1 Receptor”[tiab] OR “PD-1 Receptors”[tiab] OR “CD279 Antigen”[tiab] OR “CD279 Antigens”[tiab] OR “PD1 Receptor”[tiab] OR “PD1 Receptors”[tiab] OR “Programmed Cell Death 1 Protein”[tiab] OR “Programmed Cell Death 1 Proteins”[tiab] OR “antigen B7 H1”[tiab] OR “antigen B7H1”[tiab] OR “antigen CD152”[tiab] OR “antigen CD274”[tiab] OR “antigen CD279”[tiab] OR “B7 H1 antigen”[tiab] OR “B7 H1 antigens”[tiab] OR “B7 H1 protein”[tiab] OR “B7 H1 proteins”[tiab] OR “B7 homolog 1 protein “[tiab] OR “B7 homolog 1 proteins”[tiab] OR “B7H1 antigen”[tiab] OR “B7H1 antigens”[tiab] OR “B7H1 Immune Costimulatory Protein”[tiab] OR “B7-H1 Immune Costimulatory Protein”[tiab] OR “B7H1 Immune Costimulatory Proteins”[tiab] OR “B7-H1 Immune Costimulatory Proteins”[tiab] OR “B7H1 protein”[tiab] OR “B7H1 proteins”[tiab] OR “CD152 antibody”[tiab] OR “CD152 antibodies”[tiab] OR “CD152 antigen”[tiab] OR “CD152 antigens”[tiab] OR “CD274 antigen”[tiab] OR “CD274 antigens”[tiab] OR “CD279 antigen”[tiab] OR “CD279 antigens”[tiab] OR “CTLA 4 antibodies”[tiab] OR “CTLA 4 antibody”[tiab] OR “CTLA 4 antigen”[tiab] OR “CTLA 4 antigens”[tiab] OR “CTLA4 antibodies”[tiab] OR “CTLA4 antibody”[tiab] OR “CTLA4 antigen”[tiab] OR “CTLA4 antigens”[tiab] OR “cytotoxic T lymphocyte antigen 4”[tiab] OR “cytotoxic T lymphocyte antigen 4 antibodies”[tiab] OR “cytotoxic T lymphocyte antigen 4 antibody”[tiab] OR “cytotoxic T lymphocyte associated antigen 4”[tiab] OR “Cytotoxic T-Lymphocyte Antigen 4”[tiab] OR “Cytotoxic T-Lymphocyte-Associated Antigen 4”[tiab] OR “PD 1 protein”[tiab] OR “PD 1 proteins”[tiab] OR “PD1 protein”[tiab] OR “PD1 proteins”[tiab] OR “PD1 receptor”[tiab] OR “PD1 receptors”[tiab] OR “PD-1 receptor”[tiab] OR “PD-1 receptors”[tiab] OR “PDCD1 ligand 1”[tiab] OR “PDCD1 protein”[tiab] OR “PDCD1 proteins”[tiab] OR “PDCD1LG1 protein”[tiab] OR “PDCD1LG1 proteins”[tiab] OR “PD-L1 Costimulatory Protein”[tiab] OR “PD-L1 Costimulatory Proteins”[tiab] OR “programmed cell death 1 ligand 1”[tiab] OR “Programmed Cell Death 1 Ligand 1 Protein”[tiab] OR “Programmed Cell Death 1 Ligand 1 Proteins”[tiab] OR “programmed cell death 1 protein”[tiab] OR “programmed cell death 1 Proteins”[tiab] OR “programmed cell death 1 receptor”[tiab] OR “programmed cell death 1 receptors”[tiab] OR “programmed cell death protein 1”[tiab] OR “programmed death 1 ligand 1”[tiab] OR “programmed death 1 ligand 1 protein”[tiab] OR “programmed death 1 ligand 1 proteins”[tiab] OR “programmed death 1 protein”[tiab] OR “programmed death 1 proteins”[tiab] OR “programmed death 1 receptor”[tiab] OR “programmed death 1 receptors”[tiab] OR “programmed death ligand 1”[tiab] OR “programmed death protein 1”[tiab] OR “protein B7 H1”[tiab] OR “protein B7H1”[tiab] OR “protein PD1”[tiab] OR “protein PD 1”[tiab] OR “protein PDCD1”[tiab] OR “protein PDCD1LG1”[tiab] OR “protein programmed cell death 1”[tiab] OR “protein programmed death 1”[tiab]	**15963**
**#10**	"nivolumab" [Supplementary Concept]) OR "pembrolizumab" [Supplementary Concept]) OR "Ipilimumab"[Mesh] OR "durvalumab" [Supplementary Concept] OR Nivolumab*[tiab] OR "bms 936558"[tiab] OR bms936558[tiab] OR "mdx 1106"[tiab] OR mdx1106[tiab] OR "ono 4538"[tiab] OR ono4538[tiab] OR opdivo[tiab] OR	**7685**

	Pembrolizumab*[tiab] OR keytruda[tiab] OR lambrolizumab*[tiab] OR "mk 3475"[tiab] OR mk3475[tiab] OR Ipilimumab*[tiab] OR "bms 734016"[tiab] OR bms734016[tiab] OR "mdx 010"[tiab] OR “mdx 101”[tiab] OR mdx010[tiab] OR mdx101[tiab] OR strentarga[tiab] OR yervoy[tiab] OR Durvalumab*[tiab] OR imfinzi[tiab] OR 'medi 4736'[tiab] OR medi4736[tiab]	
**#9**	"Immunotherapy"[Mesh:NoExp] OR "Immunization"[Mesh] OR Immunotherap*[tiab] OR Immunization*[tiab] OR Immunisation*[tiab]	**314144**
**#8**	#6 AND #7	**59524**
**#7**	"Neoplasm Metastasis"[Mesh] OR metasta*[tiab] OR advanced[tiab] OR "secondary" [Subheading] OR secondary[tiab]	**1533583**
**#6**	#2 OR #5	**474860**
**#5**	#3 AND #4	**474860**
**#4**	"Brain"[Mesh] OR "Brain Diseases"[Mesh] OR brain*[tiab] OR supratentori*[tiab] OR periventricul*[tiab] OR cerebr*[tiab] OR cerebe*[tiab] OR cranial*[tiab] OR white matter[tiab] OR basal gangli*[tiab] OR thalam*[tiab] OR nucleus caudat*[tiab]	**2586863**
**#3**	"Neoplasms"[Mesh] OR cancer[sb] OR adenoma*[tw] OR anticarcinogen*[tw] OR blastoma*[tw] OR cancer*[tw] OR carcinogen*[tw] OR carcinom*[tw] OR carcinosarcoma*[tw] OR chordoma*[tw] OR germinoma*[tw] OR gonadoblastoma*[tw] OR hepatoblastoma*[tw] OR hodgkin disease[tw] OR hodgkin's disease[tw] OR hodgkins disease[tw] OR leukemi*[tw] OR lymphangioma*[tw] OR lymphangiomyoma*[tw] OR lymphangiosarcoma*[tw] OR lymphom*[tw] OR malignan*[tw] OR melanom*[tw] OR meningioma*[tw] OR mesenchymoma*[tw] OR mesonephroma*[tw] OR metasta*[tw] OR neoplas*[tw] OR neuroma*[tw] OR nsclc[tw] OR oncogen*[tw] OR oncolog*[tw] OR paraneoplastic[tw] OR plasmacytoma*[tw] OR precancerous[tw] OR sarcoma*[tw] OR teratocarcinoma*[tw] OR teratoma*[tw] OR tumor*[tw] OR tumour*[tw]	**6201757**
**#2**	"Brain Neoplasms"[Mesh]	**147383**
**#1**	"Glioblastoma"[Mesh] OR glioblastoma*[tiab] OR Grade IV Astrocytoma*[tiab] OR GBM*[tiab]	**44189**

Search strategy in Embase.com (2019 November 11th)

#	**Query**	**Results**
**#15**	#13 OR #14	**10234**
**#14**	#8 AND #12	**5675**
**#13**	#1 AND #12	**5045**
**#12**	#9 OR #10 OR #11	**566121**

**#11**	'cytotoxic t lymphocyte antigen 4'/exp OR 'cytotoxic t lymphocyte antigen 4 antibody'/exp OR 'programmed death 1 receptor'/exp OR 'programmed death 1 ligand 1'/exp OR 'antigen b7 h1':ti,ab,kw OR 'antigen b7h1':ti,ab,kw OR 'antigen cd152':ti,ab,kw OR 'antigen cd274':ti,ab,kw OR 'antigen cd279':ti,ab,kw OR 'b7 h1 antigen':ti,ab,kw OR 'b7 h1 antigens':ti,ab,kw OR 'b7 h1 protein':ti,ab,kw OR 'b7 h1 proteins':ti,ab,kw OR 'b7 homolog 1 protein':ti,ab,kw OR 'b7 homolog 1 proteins':ti,ab,kw OR 'b7h1 antigen':ti,ab,kw OR 'b7h1 antigens':ti,ab,kw OR 'b7h1 immune costimulatory protein':ti,ab,kw OR 'b7-h1 immune costimulatory protein':ti,ab,kw OR 'b7h1 immune costimulatory proteins':ti,ab,kw OR 'b7-h1 immune costimulatory proteins':ti,ab,kw OR 'b7h1 protein':ti,ab,kw OR 'b7h1 proteins':ti,ab,kw OR 'cd152 antibody':ti,ab,kw OR 'cd152 antibodies':ti,ab,kw OR 'cd152 antigen':ti,ab,kw OR 'cd152 antigens':ti,ab,kw OR 'cd274 antigen':ti,ab,kw OR 'cd274 antigens':ti,ab,kw OR 'cd279 antigen':ti,ab,kw OR 'cd279 antigens':ti,ab,kw OR 'ctla 4 antibodies':ti,ab,kw OR 'ctla 4 antibody':ti,ab,kw OR 'ctla 4 antigen':ti,ab,kw OR 'ctla 4 antigens':ti,ab,kw OR 'ctla4 antibodies':ti,ab,kw OR 'ctla4 antibody':ti,ab,kw OR 'ctla4 antigen':ti,ab,kw OR 'ctla4 antigens':ti,ab,kw OR 'cytotoxic t lymphocyte antigen 4':ti,ab,kw OR 'cytotoxic t lymphocyte antigen 4 antibodies':ti,ab,kw OR 'cytotoxic t lymphocyte antigen 4 antibody':ti,ab,kw OR 'cytotoxic t lymphocyte associated antigen 4':ti,ab,kw OR 'cytotoxic t-lymphocyte antigen 4':ti,ab,kw OR 'cytotoxic t-lymphocyte-associated antigen 4':ti,ab,kw OR 'pd 1 protein':ti,ab,kw OR 'pd 1 proteins':ti,ab,kw OR 'pd1 protein':ti,ab,kw OR 'pd1 proteins':ti,ab,kw OR 'pd1 receptor':ti,ab,kw OR 'pd1 receptors':ti,ab,kw OR 'pd-1 receptor':ti,ab,kw OR 'pd-1 receptors':ti,ab,kw OR 'pdcd1 ligand 1':ti,ab,kw OR 'pdcd1 protein':ti,ab,kw OR 'pdcd1 proteins':ti,ab,kw OR 'pdcd1lg1 protein':ti,ab,kw OR 'pdcd1lg1 proteins':ti,ab,kw OR 'pd-l1 costimulatory protein':ti,ab,kw OR 'pd-l1 costimulatory proteins':ti,ab,kw OR 'programmed cell death 1 ligand 1':ti,ab,kw OR 'programmed cell death 1 ligand 1 protein':ti,ab,kw OR 'programmed cell death 1 ligand 1 proteins':ti,ab,kw OR 'programmed cell death 1 protein':ti,ab,kw OR 'programmed cell death 1 proteins':ti,ab,kw OR 'programmed cell death 1 receptor':ti,ab,kw OR 'programmed cell death 1 receptors':ti,ab,kw OR 'programmed cell death protein 1':ti,ab,kw OR 'programmed death 1 ligand 1':ti,ab,kw OR 'programmed death 1 ligand 1 protein':ti,ab,kw OR 'programmed death 1 ligand 1 proteins':ti,ab,kw OR 'programmed death 1 protein':ti,ab,kw OR 'programmed death 1 proteins':ti,ab,kw OR 'programmed death 1 receptor':ti,ab,kw OR 'programmed death 1 receptors':ti,ab,kw OR 'programmed death ligand 1':ti,ab,kw OR 'programmed death protein 1':ti,ab,kw OR 'protein b7 h1':ti,ab,kw OR 'protein b7h1':ti,ab,kw OR 'protein pd1':ti,ab,kw OR 'protein pd 1':ti,ab,kw OR 'protein pdcd1':ti,ab,kw OR 'protein pdcd1lg1':ti,ab,kw OR 'protein programmed cell death 1':ti,ab,kw OR 'protein programmed death 1':ti,ab,kw	**47833**
**#10**	'nivolumab'/exp OR 'pembrolizumab'/exp OR 'ipilimumab'/exp OR 'durvalumab'/exp OR nivolumab*:ti,ab,kw OR 'bms 936558':ti,ab,kw OR bms936558:ti,ab,kw OR 'mdx 1106':ti,ab,kw OR mdx1106:ti,ab,kw OR 'ono 4538':ti,ab,kw OR ono4538:ti,ab,kw OR opdivo:ti,ab,kw OR pembrolizumab*:ti,ab,kw OR keytruda:ti,ab,kw OR lambrolizumab*:ti,ab,kw OR 'mk 3475':ti,ab,kw OR mk3475:ti,ab,kw OR ipilimumab*:ti,ab,kw OR 'bms 734016':ti,ab,kw OR bms734016:ti,ab,kw OR 'mdx 010':ti,ab,kw OR 'mdx 101':ti,ab,kw OR mdx010:ti,ab,kw OR mdx101:ti,ab,kw OR strentarga:ti,ab,kw OR yervoy:ti,ab,kw OR durvalumab*:ti,ab,kw OR imfinzi:ti,ab,kw OR 'medi 4736':ti,ab,kw OR medi4736:ti,ab,kw	**26672**
**#9**	'immunotherapy'/de OR 'active immunotherapy'/exp OR 'adoptive immunotherapy'/exp OR 'cancer immunotherapy'/de OR 'cancer immunization'/exp OR 'immunopotentiation'/exp OR 'oral immunotherapy'/exp OR 'subcutaneous immunotherapy'/exp OR 'sublingual immunotherapy'/exp OR 'immunization'/exp OR immunotherap*:ti,ab,kw OR immunization*:ti,ab,kw OR immunisation*:ti,ab,kw	**529452**
**#8**	#6 AND #7	**102801**
**#7**	'metastasis'/exp OR metasta*:ab,ti,kw OR advanced:ab,ti,kw OR secondary:ti,ab,kw	**2194279**
**#6**	#2 OR #5	**491838**
**#5**	#3 AND #4	**491838**
**#4**	'brain'/exp OR 'brain disease'/exp OR brain*:ti,ab,kw OR supratentori*:ti,ab,kw OR periventricul*:ti,ab,kw OR cerebr*:ti,ab,kw OR cranial*:ti,ab,kw OR 'white matter':ti,ab,kw OR ((basal NEAR/2 gangli*):ti,ab,kw) OR thalam*:ti,ab,kw OR ((nucleus NEAR/2 caudat*):ti,ab,kw)	**3607343**

#3	'neoplasm'/exp OR adenoma*:ti,ab,kw OR anticarcinogen*:ti,ab,kw OR blastoma*:ti,ab,kw OR cancer*:ti,ab,kw OR carcinogen*:ti,ab,kw OR carcinom*:ti,ab,kw OR carcinosarcoma*:ti,ab,kw OR chordoma*:ti,ab,kw OR germinoma*:ti,ab,kw OR gonadoblastoma*:ti,ab,kw OR hepatoblastoma*:ti,ab,kw OR ((hodgkin* NEXT/1 disease):ti,ab,kw) OR leukemi*:ti,ab,kw OR lymphangioma*:ti,ab,kw OR lymphangiomyoma*:ti,ab,kw OR lymphangiosarcoma*:ti,ab,kw OR lymphom*:ti,ab,kw OR malignan*:ti,ab,kw OR melanom*:ti,ab,kw OR meningioma*:ti,ab,kw OR mesenchymoma*:ti,ab,kw OR mesonephroma*:ti,ab,kw OR metasta*:ti,ab,kw OR neoplas*:ti,ab,kw OR neuroma*:ti,ab,kw OR nsclc:ti,ab,kw OR oncogen*:ti,ab,kw OR oncolog*:ti,ab,kw OR paraneoplastic:ti,ab,kw OR plasmacytoma*:ti,ab,kw OR precancerous:ti,ab,kw OR sarcoma*:ti,ab,kw OR teratocarcinoma*:ti,ab,kw OR teratoma*:ti,ab,kw OR tumor*:ti,ab,kw OR tumour*:ti,ab,kw	**5814105**
#2	'brain tumor'/exp	**183115**
#1	'glioblastoma'/exp OR glioblastoma*:ti,ab,kw OR 'grade iv astrocytoma*':ti,ab,kw OR gbm*:ti,ab,kw	**80115**

Search strategy in the Cochrane Library via Wiley (2019 November 11th)

#	Query	Results
#13	#11 or #12	**505**
#12	#6 and #10	**330**
#11	#1 and #10	**200**
#10	#7 or #8 or #9	**19928**
#9	“antigen b7 h1”:ti,ab,kw or “antigen b7h1”:ti,ab,kw or “antigen cd152”:ti,ab,kw or “antigen cd274”:ti,ab,kw or “antigen cd279”:ti,ab,kw or “b7 h1 antigen”:ti,ab,kw or “b7 h1 antigens”:ti,ab,kw or “b7 h1 protein”:ti,ab,kw or “b7 h1 proteins”:ti,ab,kw or “b7 homolog 1 protein”:ti,ab,kw or “b7 homolog 1 proteins”:ti,ab,kw or “b7h1 antigen”:ti,ab,kw or “b7h1 antigens”:ti,ab,kw or “b7h1 immune costimulatory protein”:ti,ab,kw or “b7 h1 immune costimulatory protein”:ti,ab,kw or “b7h1 immune costimulatory proteins”:ti,ab,kw or “b7 h1 immune costimulatory proteins”:ti,ab,kw or “b7h1 protein”:ti,ab,kw or “b7h1 proteins”:ti,ab,kw or “cd152 antibody”:ti,ab,kw or “cd152 antibodies”:ti,ab,kw or “cd152 antigen”:ti,ab,kw or “cd152 antigens”:ti,ab,kw or “cd274 antigen”:ti,ab,kw or “cd274 antigens”:ti,ab,kw or “cd279 antigen”:ti,ab,kw or “cd279 antigens”:ti,ab,kw or “ctla 4 antibodies”:ti,ab,kw or “ctla 4 antibody”:ti,ab,kw or “ctla 4 antigen”:ti,ab,kw or “ctla 4 antigens”:ti,ab,kw or “ctla4 antibodies”:ti,ab,kw or “ctla4 antibody”:ti,ab,kw or “ctla4 antigen”:ti,ab,kw or “ctla4 antigens”:ti,ab,kw or “cytotoxic t lymphocyte antigen 4”:ti,ab,kw or “cytotoxic t lymphocyte antigen 4 antibodies”:ti,ab,kw or “cytotoxic t lymphocyte antigen 4 antibody”:ti,ab,kw or “cytotoxic t lymphocyte associated antigen 4”:ti,ab,kw or “cytotoxic t-lymphocyte antigen 4”:ti,ab,kw or “cytotoxic t-lymphocyte-associated antigen 4”:ti,ab,kw or “pd 1 protein”:ti,ab,kw or “pd 1 proteins”:ti,ab,kw or “pd1 protein”:ti,ab,kw or “pd1 proteins”:ti,ab,kw or “pd1 receptor”:ti,ab,kw or “pd1 receptors”:ti,ab,kw or “pd-1 receptor”:ti,ab,kw or “pd-1 receptors”:ti,ab,kw or “pdcd1 ligand 1”:ti,ab,kw or “pdcd1 protein”:ti,ab,kw or “pdcd1 proteins”:ti,ab,kw or “pdcd1lg1 protein”:ti,ab,kw or “pdcd1lg1 proteins”:ti,ab,kw or “pd-l1 costimulatory protein”:ti,ab,kw or “pd-l1 costimulatory proteins”:ti,ab,kw or “programmed cell death 1 ligand 1”:ti,ab,kw or “programmed cell death 1 ligand 1 protein”:ti,ab,kw or “programmed cell death 1 ligand 1 proteins”:ti,ab,kw or “programmed cell death 1 protein”:ti,ab,kw or “programmed cell death 1 proteins”:ti,ab,kw or “programmed cell death 1 receptor”:ti,ab,kw or “programmed cell death 1 receptors”:ti,ab,kw or “programmed cell death protein 1”:ti,ab,kw or “programmed death 1 ligand 1”:ti,ab,kw or “programmed death 1 ligand 1 protein”:ti,ab,kw or “programmed death 1 ligand 1 proteins”:ti,ab,kw or “programmed death 1 protein”:ti,ab,kw or “programmed death 1 proteins”:ti,ab,kw or “programmed death 1 receptor”:ti,ab,kw or “programmed death 1 receptors”:ti,ab,kw or “programmed death ligand 1”:ti,ab,kw or “programmed death protein 1”:ti,ab,kw	**1126**

	or "protein b7 h1":ti,ab,kw or "protein b7h1":ti,ab,kw or "protein pd1":ti,ab,kw or "protein pd 1":ti,ab,kw or "protein pdcd1":ti,ab,kw or "protein pdcd1lg1":ti,ab,kw or "protein programmed cell death 1":ti,ab,kw or "protein programmed death 1":ti,ab,kw	
#8	Nivolumab*:ti,ab,kw or "bms 936558":ti,ab,kw or bms936558:ti,ab,kw or "mdx 1106":ti,ab,kw or mdx1106:ti,ab,kw or "ono 4538":ti,ab,kw or ono4538:ti,ab,kw or opdivo:ti,ab,kw or pembrolizumab*:ti,ab,kw or keytruda:ti,ab,kw or lambrolizumab*:ti,ab,kw or "mk 3475":ti,ab,kw or mk3475:ti,ab,kw or ipilimumab*:ti,ab,kw or "bms 734016":ti,ab,kw or bms734016:ti,ab,kw or "mdx 010":ti,ab,kw or "mdx 101":ti,ab,kw or mdx010:ti,ab,kw or mdx101:ti,ab,kw or strentarga:ti,ab,kw or yervoy:ti,ab,kw or durvalumab*:ti,ab,kw or imfinzi:ti,ab,kw or "medi 4736":ti,ab,kw or medi4736:ti,ab,kw	**3117**
#7	immunotherap*:ti,ab,kw or immunization*:ti,ab,kw or immunisation*:ti,ab,kw	**17221**
#6	#4 and #5	**4713**
#5	metasta*:ab,ti,kw or advanced:ab,ti,kw or secondary:ti,ab,kw	**286976**
#4	#2 and #3	**10449**
#3	brain*:ti,ab,kw or supratentori*:ti,ab,kw or periventricul*:ti,ab,kw or cerebr*:ti,ab,kw or cranial*:ti,ab,kw or "white matter":ti,ab,kw or ((basal gangli*):ti,ab,kw) or thalam*:ti,ab,kw or ((nucleus caudat*):ti,ab,kw)	**89456**
#2	adenoma*:ti,ab,kw or anticarcinogen*:ti,ab,kw or blastoma*:ti,ab,kw or cancer*:ti,ab,kw or carcinogen*:ti,ab,kw or carcinom*:ti,ab,kw or carcinosarcoma*:ti,ab,kw or chordoma*:ti,ab,kw or germinoma*:ti,ab,kw or gonadoblastoma*:ti,ab,kw or hepatoblastoma*:ti,ab,kw or ((hodgkin* NEXT/1 disease):ti,ab,kw) or leukemi*:ti,ab,kw or lymphangioma*:ti,ab,kw or lymphangiomyoma*:ti,ab,kw or lymphangiosarcoma*:ti,ab,kw or lymphom*:ti,ab,kw or malignan*:ti,ab,kw or melanom*:ti,ab,kw or meningioma*:ti,ab,kw or mesenchymoma*:ti,ab,kw or mesonephroma*:ti,ab,kw or metasta*:ti,ab,kw or neoplas*:ti,ab,kw or neuroma*:ti,ab,kw or nsclc:ti,ab,kw or oncogen*:ti,ab,kw or oncolog*:ti,ab,kw or paraneoplastic:ti,ab,kw or plasmacytoma*:ti,ab,kw or precancerous:ti,ab,kw or sarcoma*:ti,ab,kw or teratocarcinoma*:ti,ab,kw or teratoma*:ti,ab,kw or tumor*:ti,ab,kw or tumour*:ti,ab,kw	**224019**
#1	glioblastoma*:ti,ab,kw or "grade iv astrocytoma*":ti,ab,kw or gbm*:ti,ab,kw	**2113**

Table S2. Studies with immune checkpoint inhibitors in patients with brain metastases that only reported overall survival data.

**Author**	**Study Design**	**Tumor Type**	**Agent**	**No. of Patients**	**OS**
					Months
Ahmad (2015) [1]	EAP (Retrospective)	Melanoma	IPI	35	3.5 (95% CI 2.5–4.9)
Alexander (2014) [2]	Retrospective	Melanoma	IPI	44	9.3 (95% CI 4.9–12.4)
Ascierto (2017) Cohort A [3]	III	Melanoma	IPI	65	7.0 (95% CI 4.0–12.8)
Ascierto (2017) Cohort B	III	Melanoma	IPI	62	5.7 (95% CI 4.2–7.0)
Dagogo-Jack (2017) [4]	Retrospective	Melanoma	PEMBRO	36	20.4 (95% CI 15.0–N.R.)
Forschner (2017) [5]	Real-world	Melanoma	N.A.	10	7.0 (95% CI 0.0–17.9)
Jinga (2018) [6]	EAP	Melanoma	IPI	16	3.9 (95% CI 0.0–9.2)
Larkin (2018) [7]	III	Melanoma	NIVO	55	8.7 (95% CI 6.3–13.7)
Liu (2019) [8]	Retrospective	Melanoma	PEMBRO	96	18.4 (95% CI 10.8–25.1)
Mangana (2017) [9]	Retrospective	Melanoma	IPI or anti-PD-1	14	10.9

Milsch (2018) [10]	Retrospective	Melanoma	IPI, NIVO or PEMBRO	177	10.5 (95% CI 8.6–12.4)
Schvartsman (2019) [11]	Retrospective	Melanoma	NIVO or PEMBRO	75	32.2 (95% CI 22.9–N.R.)
Vosoughi (2018) [12]	Retrospective	Melanoma	anti-CTLA-4	39	19.2 (95% CI 1.2–65.0)
Vosoughi (2018)	Retrospective	Melanoma	anti-PD-1	28	37.9 (95% CI 5.3–65.0)
Juergens (2018) [13]	Retrospective	NSCLC	NIVO	62	9.0 (95% CI 5.5–13.3)

EAP Expanded access program; *ICI* Immune checkpoint inhibitor; *IPI* Ipilimumab; *N.A.* Not available; *NIVO* Nivolumab; *N.R.* Not reached; *PEMBRO* Pembrolizumab.

Table S3. Primary outcomes of clinical studies with immune checkpoint inhibitors and radiotherapy in patients with brain metastases.

Author	Study Design	Tumor Type	Agent	Type of Radiation Therapy	No. of Patients	Local BM Control	Distant BM Control	PFS	OS
						% at 12 months	% at 12 months	Months	Months
Acharya (2017) [14]	Retrospective	Melanoma	Anti-PD-1 and/or Anti-CTLA-4	SRS within 3 months	18	85.0	60.0	N.A.	N.A.
Ahmed (2016) anti-PD-1 [15]	Retrospective	Melanoma	NIVO PEMBRO	SRS within 3 months	21	83.0	38.0	3.4	8.9
Ahmed (2016) anti-CTL-4	Retrospective	Melanoma	IPI	SRS within 3 months	25	83.0	21.0	3.4	8.9
An (2017) Cohort A1 [16]	Retrospective	Melanoma	IPI	SRS within 5.5 months	51	51.0	N.A.	8.4†	13.4
An (2017) Cohort A2	Retrospective	Melanoma	IPI	SRS after 5.5. months	20	25.0	N.A.	3.6†	11.5
An (2017) Cohort B1	Retrospective	Melanoma	IPI	SRS within 5.5 months	15	46.2	N.A.	6.3†	13.4
An (2017) Cohort B2	Retrospective	Melanoma	IPI	SRS after 5.5. months	13	20.6	N.A.	3.5†	11.5
Choong (2017) anti-PD-1 [17]	Retrospective	Melanoma	Anti-PD-1	SRS	11	48.0	N.A.	12.7† (95% CI 5.5–N.A.)	20.4 (95% CI 8.8–N.A.)
Choong (2017) anti-CTLA-4	Retrospective	Melanoma	Anti-CTLA-4	SRS	28	48.0	N.A.	7.5† (95% CI 4.0–15.6)	7.5 (95% CI 4.4–15.6)
Cohen-Inbar (2017) Cohort A [18]	Retrospective	Melanoma	IPI	SRS prior to /concurrent with ICI	32	54.0	26.0	7.2†	13.8
Cohen-Inbar (2017) Cohort B	Retrospective	Melanoma	IPI	SRS after ICI	14	17.0	17.0	5.0†	6.4
Diao (2018) Cohort A [19]	Retrospective	Melanoma	IPI	Concurrent SRS within 1 month	23	58.0	23.0	N.A.	11.8

Diao (2018) Cohort B	Retrospective	Melanoma	IPI	Non-concurrent SRS after 1 month	28	70.0	45.0	N.A.	18.7
Gerber (2015) [20]	Retrospective	Melanoma	IPI	WBRT	13	N.A.	N.A.	N.A.	4.0
Kaidar-Person (2017) [21]	Retrospective	Melanoma	IPI PEMBRO NIVO	SRS or HFRT	29	52.0	35.0	3.5†	15.0 (95% CI 10.0–15.8)
Mathew (2013) [22]	Retrospective	Melanoma	IPI	SRS	25	N.A.	N.A.	N.A.	N.A.
Minniti (2019) Cohort A [23]	Retrospective	Melanoma	IPI	SRS concurrent with ICI	45	70.0	N.A.	6.0†	14.7
Minniti (2019) Cohort B	Retrospective	Melanoma	NIVO	SRS concurrent with ICI	35	85.0	N.A.	10.0†	22.0
Murphy (2019) [24]	Retrospective	Melanoma	IPI PEMBRO NIVO	SRS	26	95.4	N.A.	N.A.	26.1
Nardin (2018) [25]	Retrospective	Melanoma	PEMBRO	SRS	25	N.A.	36.0	4.0† (95% CI 2.2–5.8)	11.0 (95% CI 5.3–16.7)
Olson (2016) [26]	Retrospective	Melanoma	IPI	SRS	27	44.0	26.0	3.3 (95% CI–2.8–N.R.)	10.4 (94% CI 6.5–23.4)
Patel (2017) [27]	Retrospective	Melanoma	IPI	SRS	54	71.4	12.7	N.A.	N.A.
Robin (2018) [28]	Retrospective	Melanoma	Anti-PD-1 and/or Anti-CTLA-4	SRS	38	N.A.	N.A.	3.4	N.R.
Silk (2013) Cohort A [29]	Retrospective	Melanoma	IPI	SRS or WBRT prior to ICI	21	N.A.	N.A.	2.7† (95% CI 1.5–6.0)	18.4
Silk (2013) Cohort B	Retrospective	Melanoma	IPI	SRS or WBRT after ICI	12	N.A.	N.A.	2.7† (95% CI 1.5–6.0)	8.1
Williams (2017) Cohort A [30]	I	Melanoma	IPI	WBRT concurrent with ICI	5	N.A.	N.A.	2.5† (95% CI 0.3 –18.0)	8.0
Williams (2017) Cohort B	I	Melanoma	IPI	SRS concurrent with ICI	11	N.A.	N.A.	2.5† (95% CI 1.0–37.0)	N.R.
Yusuf (2017) [31]	Retrospective	Melanoma	Anti-PD-1 and/or Anti-CTLA-4	SRS concurrent with ICI	18	N.A.	N.A.	N.A.	7.4
Chen (2018) Cohort A [32]	Retrospective	Melanoma NSCLC RCC	Anti-PD-1 and/or	Concurrent SRS-SRT	28	88.0	N.A.	11.5†	24.7

			Anti-CTLA-4						
Chen (2018) Cohort B	Retrospective	Melanoma NSCLC RCC	Anti-PD-1 and/or Anti-CTLA-4	Non-concurrent SRS-SRT	51	79.0	N.A.	5.1†	14.5
Lanier (2019) [33]	Retrospective	Melanoma NSCLC	Anti-PD-1 and/or Anti-CTLA-4	SRS	101	91.0	46.0	N.A.	15.9 (95% CI 13.3–24.8)
Schapira (2018) Cohort A [34]	Retrospective	NSCLC	Anti-PD-1	SRS prior to ICI	24	72.3	34.2	N.A.	N.A.
Schapira (2018) Cohort B	Retrospective	NSCLC	Anti-PD-1	SRS concurrent with ICI	8	100.0	61.5	N.A.	N.A.
Schapira (2018) Cohort C	Retrospective	NSCLC	Anti-PD-1	SRS after ICI	5	100.0	0.0	N.A.	N.A.
Shepard (2019) Cohort A [35]	Retrospective	NSCLC	NIVO or PEMBRO	SRS within 5 months	17	100.0	47.5	N.A.	N.R.
Shepard (2019) Cohort B	Retrospective	NSCLC	NIVO or PEMBRO	Non-concurrent SRS	34	85.2	66.5	5.5†	15.9
Koenig (2019) Cohort A [36]	Retrospective	N.A.	Anti-PD-1 and/or Anti-CTLA-4	SRS within 5 months	70	95.9	33.8	N.A.	11.3
Koenig (2019) Cohort B	Retrospective	N.A.	Anti-PD-1 and/or Anti-CTLA-4	Non-concurrent SRS	27	97.1	28.0	N.A.	5.9

* Intracranial ORR; †Intracranial PFS. *EAP* Expanded access program; *ICI* Immune checkpoint inhibitor; *IPI* Ipilimumab; *N.A.* Not available; *NIVO* Nivolumab; *N.R.* Not reached; *PEMBRO* Pembrolizumab

Table S4. Clinical studies with immune checkpoint inhibitors and radiotherapy in patients with brain metastases that only reported overall survival data.

Author	Study design	Tumor type	Agent	Type of radiation therapy	No. of patients	OS
						Months
Amaral (2019)[37]	Retrospective	Melanoma	Anti-PD-1 and/or Anti-CTLA-4	SRS	36	25.0 (95% CI 14.6–35.4)
Anderson (2017) Cohort A [38]	Retrospective	Melanoma	PEMBRO	SRS, HFRT or WBRT within 4 months	21	N.A.
Anderson (2017) Cohort B	Retrospective	Melanoma	IPI	SRS, HFRT or WBRT within 4 months	20	N.A.

Gabani (2018) Cohort A [39]	Retrospective	Melanoma	IPI	SRS	89	17.0 (95% CI 10.7–23.2)
Gabani (2018) Cohort B	Retrospective	Melanoma	IPI	WBRT	103	8.5 (95% CI 6.5–10.5)
Galli (2019) Cohort A [40]	Retrospective	Melanoma	IPI	SRS or WBRT within 6 months	23	5.0
Galli (2019) Cohort A	Retrospective	Melanoma	NIVO or PEMBRO	SRS or WBRT within 6 months	13	18.0
Kiess (2015) Cohort A [41]	Retrospective	Melanoma	IPI	SRS prior to ICI	19	12.4 [range 2.0–89.0]
Kiess (2015) Cohort B	Retrospective	Melanoma	IPI	SRS concurrent with ICI	15	12.4 [range 2.0–89.0]
Kiess (2015) Cohort C	Retrospective	Melanoma	IPI	SRS after ICI	12	12.4 [range 2.0–89.0]
Knisely (2012) Cohort A [42]	Retrospective	Melanoma	IPI	SRS prior to ICI	16	21.3 (95% CI 15.7–N.R.)
Knisely (2012) Cohort B	Retrospective	Melanoma	IPI	SRS after ICI	11	19.8 (95% CI 1.5–N.R.)
Rahman (2018) Cohort A [43]	Retrospective	Melanoma	IPI<br>PEMBRO<br>NIVO	Concurrent SRS within 1 month	35	13.9
Rahman (2018) Cohort B	Retrospective	Melanoma	IPI<br>PEMBRO<br>NIVO	Non-concurrent SRS	39	13.9
Rauschenberg (2019) Cohort A [44]	Retrospective	Melanoma	Anti-PD-1 and/or Anti-CTLA-4	SRS	87	21.0 (95% CI 12.9–29.1)
Rauschenberg (2019) Cohort A1	Retrospective	Melanoma	Anti-PD-1 and/or Anti-CTLA-4	SRS prior to ICI	26	17.6 (95% CI 10.3–25.0)
Rauschenberg (2019) Cohort A2	Retrospective	Melanoma	Anti-PD-1 and/or Anti-CTLA-4	SRS concurrent with ICI	49	N.R.
Rauschenberg (2019) Cohort A3	Retrospective	Melanoma	Anti-PD-1 and/or Anti-CTLA-4	SRS after ICI	12	21.9 (95% CI 10.4–33.4)
Rauschenberg (2019) Cohort B	Retrospective	Melanoma	Anti-PD-1 and/or Anti-CTLA-4	WBRT	51	7.1 (95% CI 5.1–9.1)
Rauschenberg (2019) Cohort B1	Retrospective	Melanoma	Anti-PD-1 and/or Anti-CTLA-4	WBRT prior to ICI	21	8.9 (95% CI 6.0–11.8)
Rauschenberg (2019) Cohort B2	Retrospective	Melanoma	Anti-PD-1 and/or Anti-CTLA-4	WBRT concurrent with ICI	22	6.7 (95% CI 3.3–10.1)
Rauschenberg (2019) Cohort B3	Retrospective	Melanoma	Anti-PD-1 and/or Anti-CTLA-4	WBRT after ICI	8	2.4 (95% CI 0.0–9.4)
Schmidberger (2018) Cohort A [45]	Retrospective	Melanoma	IPI	SRS or WBRT prior to ICI	21	11.0
Schmidberger (2018) Cohort B	Retrospective	Melanoma	IPI	SRS or WBRT after ICI	20	3.0
Stokes (2017)	Retrospective	Melanoma	Anti-PD-1 and/or Anti-CTLA-4	SRS	185	10.8 (95% CI 9.1–12.5)
Tazi (2015) Cohort A [46]	Retrospective	Melanoma	IPI	SRS	10	16.5 (95% CI 10.4–N.A.)
Tétu (2019) [47]	Retrospective	Melanoma	Anti-PD-1 and/or Anti-CTLA-4	Concurrent SRS	64	16.8
Trommer-Nestler (2018) [48]	Retrospective	Melanoma	Anti-PD-1	Concurrent SRS	13	N.A.
Ahmed (2017) [49]	Retrospective	NSCLC	NIVO<br>DUR	SRS	17	5.6

Singh (2019) [50]	Retrospective	NSCLC	PEMBRO<br>NIVO<br>ATEZO	SRS	39	10.0 (95% CI 8.3–13.2)
Kotecha (2019) [51]	Retrospective	N.A.	Anti-PD-1 and/or Anti-CTLA-4	Concurrent and non-concurrent SRS	150	30.0 (95% CI 24.0–38.0)
Shen (2016) [52]	Retrospective	N.A.	Anti-PD-1 and/or Anti-CTLA-4	Concurrent SRS	20	5.5 (95% CI 1.8–9.2)

EAP Expanded access program; *ICI* Immune checkpoint inhibitor; *IPI* Ipilimumab; *N.A.* Not available; *NIVO* Nivolumab; *N.R.* Not reached; *PEMBRO* Pembrolizumab.

References

1. Ahmad, S.S.; Qian, W.; Ellis, S.; Mason, E.; Khattak, M.A.; Gupta, A.; Shaw, H.; Quinton, A.; Kovarikova, J.; Thillai, K.; et al. Ipilimumab in the real world: the UK expanded access programme experience in previously treated advanced melanoma patients. *Melanoma Res.* **2015**, *25*, 432–442.
2. Alexander, M.; Mellor, J.D.; McArthur, G.; Kee, D. Ipilimumab in pretreated patients with unresectable or metastatic cutaneous, uveal and mucosal melanoma. *Med. J. Aust* **2014**, *201*, 49–53.
3. Ascierto, P.A.; Del Vecchio, M.; Robert, C.; Mackiewicz, A.; Chiarion-Sileni, V.; Arance, A.; Lebbe, C.; Bastholt, L.; Hamid, O.; Rutkowski, P.; et al. Ipilimumab 10 mg/kg versus ipilimumab 3 mg/kg in patients with unresectable or metastatic melanoma: a randomised, double-blind, multicentre, phase 3 trial. *Lancet Oncol.* **2017**, *18*, 611–622.
4. Dagogo-Jack, I.; Lanfranchi, M.; Gainor, J.F.; Giobbie-Hurder, A.; Lawrence, D.P.; Shaw, A.T.; Sullivan, R.J. A Retrospective Analysis of the Efficacy of Pembrolizumab in Melanoma Patients With Brain Metastasis. *J. Immunother.* **2017**, *40*, 108–113.
5. Forschner, A.; Eichner, F.; Amaral, T.; Keim, U.; Garbe, C.; Eigentler, T.K. Improvement of overall survival in stage IV melanoma patients during 2011-2014: Analysis of real-world data in 441 patients of the German Central Malignant Melanoma Registry (CMMR). *J. Cancer Res. Clin. Oncol.* **2017**, *143*, 533–540.
6. Jinga, D.C.; Ciuleanu, T.; Negru, S.; Aldea, C.; Gales, L.; Bacanu, F.; Oprean, C.; Manolache, M.; Zob, D.; Curescu, S.; et al. Effectiveness and safety profile of ipilimumab therapy in previously treated patients with unresectable or metastatic melanoma–The Romanian Patient Access Program. *J. B.U.ON.* **2018**, *22*, 1287–1295.
7. Larkin, J.; Minor, D.; D'Angelo, S.; Neyns, B.; Smylie, M.; Miller, W.H., Jr.; Gutzmer, R.; Linette, G.; Chmielowski, B.; Lao, C.D.; et al. Overall Survival in Patients With Advanced Melanoma Who Received Nivolumab Versus Investigator's Choice Chemotherapy in CheckMate 037: A Randomized, Controlled, Open-Label Phase III Trial. *J. Clin. Oncol.* **2018**, *36*, 383–390.
8. Liu, F.X.; Ou, W.; Diede, S.J.; Whitman, E.D. Real-world experience with pembrolizumab in patients with advanced melanoma: A large retrospective observational study. *Medicine (Baltimore)* **2019**, *98*, e16542.
9. Mangana, J.; Cheng, P.F.; Kaufmann, C.; Amann, V.C.; Frauchiger, A.L.; Stogner, V.; Held, U.; von Moos, R.; Michielin, O.; Braun, R.P.; et al. Multicenter, real-life experience with checkpoint inhibitors and targeted therapy agents in advanced melanoma patients in Switzerland. *Melanoma Res.* **2017**, *27*, 358–368.
10. Milsch, L.; Gesierich, A.; Kreft, S.; Livingstone, E.; Zimmer, L.; Goebeler, M.; Schadendorf, D.; Schilling, B. Patterns of disease control and survival in patients with melanoma brain metastases undergoing immune-checkpoint blockade. *Eur. J. Cancer* **2018**, *99*, 58–65.
11. Schvartsman, G.; Ma, J.; Bassett, R.L., Jr.; Haydu, L.E.; Amaria, R.N.; Hwu, P.; Wong, M.K.; Hwu, W.J.; Diab, A.; Patel, S.P.; et al. Incidence, patterns of progression, and outcomes of preexisting and newly discovered brain metastases during treatment with anti-PD-1 in patients with metastatic melanoma. *Cancer* **2019**, *125*, 4193–4202.
12. Vosoughi, E.; Lee, J.M.; Miller, J.R.; Nosrati, M.; Minor, D.R.; Abendroth, R.; Lee, J.W.; Andrews, B.T.; Leng, L.Z.; Wu, M.; et al. Survival and clinical outcomes of patients with melanoma brain metastasis in the era of checkpoint inhibitors and targeted therapies. *BMC Cancer* **2018**, *18*, e490.
13. Juergens, R.A.; Mariano, C.; Jolivet, J.; Finn, N.; Rothenstein, J.; Reaume, M.N.; Faghih, A.; Labbe, C.; Owen, S.; Shepherd, F.A.; et al. Real-world benefit of nivolumab in a Canadian non-small-cell lung cancer cohort. *Curr. Oncol.* **2018**, *25*, 384–392.
14. Acharya, S.; Mahmood, M.; Mullen, D.; Yang, D.; Tsien, C.I.; Huang, J.; Perkins, S.M.; Rich, K.; Chicoine, M.; Leuthardt, E.; et al. Distant intracranial failure in melanoma brain metastases treated with stereotactic radiosurgery in the era of immunotherapy and targeted agents. *Adv. Radiat. Oncol.* **2017**, *2*, 572–580.
15. Ahmed, K.A.; Abuodeh, Y.A.; Echevarria, M.I.; Arrington, J.A.; Stallworth, D.G.; Hogue, C.; Naghavi, A.O.; Kim, S.; Kim, Y.; Patel, B.G.; et al. Clinical outcomes of melanoma brain metastases treated with stereotactic radiosurgery and anti-PD-1 therapy, anti-CTLA-4 therapy, BRAF/MEK inhibitors, BRAF inhibitor, or conventional chemotherapy. *Ann. Oncol.* **2016**, *27*, 2288–2294.
16. An, Y.; Jiang, W.; Kim, B.Y.S.; Qian, J.M.; Tang, C.; Fang, P.; Logan, J.; D'Souza, N.M.; Haydu, L.E.; Wang, X.A., et al. Stereotactic radiosurgery of early melanoma brain metastases after initiation of anti-CTLA-4 treatment is associated with improved intracranial control. *Radiother. Oncol.* **2017**, *125*, 80–88.
17. Choong, E.S.; Lo, S.; Drummond, M.; Fogarty, G.B.; Menzies, A.M.; Guminski, A.; Shivalingam, B.; Clarke, K.; Long, G.V.; Hong, A.M. Survival of patients with melanoma brain metastasis treated with stereotactic radiosurgery and active systemic drug therapies. *Eur. J. Cancer* **2017**, *75*, 169–178.
18. Cohen-Inbar, O.; Shih, H.H.; Xu, Z.; Schlesinger, D.; Sheehan, J.P. The effect of timing of stereotactic radiosurgery treatment of melanoma brain metastases treated with ipilimumab. *J. Neurosurg.* **2017**, *127*, 1007–1014.

19. Diao, K.; Bian, S.X.; Routman, D.M.; Yu, C.; Ye, J.C.; Wagle, N.A.; Wong, M.K.; Zada, G.; Chang, E.L. Stereotactic radiosurgery and ipilimumab for patients with melanoma brain metastases: clinical outcomes and toxicity. *J. Neurooncol.* **2018**, *139*, 421–429.
20. Gerber, N.K.; Young, R.J.; Barker, C.A.; Wolchok, J.D.; Chan, T.A.; Yamada, Y.; Friguglietti, L.; Beal, K. Ipilimumab and whole brain radiation therapy for melanoma brain metastases. *J. Neurooncol.* **2015**, *121*, 159–165.
21. Kaidar-Person, O.; Zagar, T.M.; Deal, A.; Moschos, S.J.; Ewend, M.G.; Sasaki-Adams, D.; Lee, C.B.; Collichio, F.A.; Fried, D.; Marks, L.B.; et al. The incidence of radiation necrosis following stereotactic radiotherapy for melanoma brain metastases: the potential impact of immunotherapy. *Anticancer Drugs* **2017**, *28*, 669–675.
22. Mathew, M.; Tam, M.; Ott, P.A.; Pavlick, A.C.; Rush, S.C.; Donahue, B.R.; Golfinos, J.G.; Parker, E.C.; Huang, P.P.; Narayana, A. Ipilimumab in melanoma with limited brain metastases treated with stereotactic radiosurgery. *Melanoma Res.* **2013**, *23*, 191–195.
23. Minniti, G.; Anzellini, D.; Reverberi, C.; Cappellini, G.C.A.; Marchetti, L.; Bianciardi, F.; Bozzao, A.; Osti, M.; Gentile, P.C.; Esposito, V. Stereotactic radiosurgery combined with nivolumab or Ipilimumab for patients with melanoma brain metastases: evaluation of brain control and toxicity. *J. Immunother. Cancer* **2019**, *7*, e102.
24. Murphy, B.; Walker, J.; Bassale, S.; Monaco, D.; Jaboin, J.; Ciporen, J.; Taylor, M.; Dai Kubicky, C. Concurrent Radiosurgery and Immune Checkpoint Inhibition: Improving Regional Intracranial Control for Patients With Metastatic Melanoma. *Am. J. Clin. Oncol.* **2019**.
25. Nardin, C.; Mateus, C.; Texier, M.; Lanoy, E.; Hibat-Allah, S.; Ammari, S.; Robert, C.; Dhermain, F. Tolerance and outcomes of stereotactic radiosurgery combined with anti-programmed cell death-1 (pembrolizumab) for melanoma brain metastases. *Melanoma Res.* **2018**.
26. Olson, A.C.; Thomas, S.; Qin, R.; Singh, B.; Salama, J.K.; Kirkpatrick, J.; Salama, A.K.S. Outcomes and toxicity of stereotactic radiosurgery for melanoma brain metastases in patients receiving ipilimumab. *Melanoma Manag.* **2016**, *3*, 177–186.
27. Patel, K.R.; Shoukat, S.; Oliver, D.E.; Chowdhary, M.; Rizzo, M.; Lawson, D.H.; Khosa, F.; Liu, Y.; Khan, M.K. Ipilimumab and Stereotactic Radiosurgery Versus Stereotactic Radiosurgery Alone for Newly Diagnosed Melanoma Brain Metastases. *Am. J. Clin. Oncol.* **2017**, *40*, 444–450.
28. Robin, T.P.; Breeze, R.E.; Smith, D.E.; Rusthoven, C.G.; Lewis, K.D.; Gonzalez, R.; Brill, A.; Saiki, R.; Stuhr, K.; Gaspar, L.E.; et al. Immune checkpoint inhibitors and radiosurgery for newly diagnosed melanoma brain metastases. *J. Neurooncol.* **2018**, *140*, 55–62.
29. Silk, A.W.; Bassetti, M.F.; West, B.T.; Tsien, C.I.; Lao, C.D. Ipilimumab and radiation therapy for melanoma brain metastases. *Cancer Med.* **2013**, *2*, 899–906.
30. Williams, N.L.; Wuthrick, E.J.; Kim, H.; Palmer, J.D.; Garg, S.; Eldredge-Hindy, H.; Daskalakis, C.; Feeney, K.J.; Mastrangelo, M.J.; Kim, L.J.; et al. Phase 1 Study of Ipilimumab Combined With Whole Brain Radiation Therapy or Radiosurgery for Melanoma Patients With Brain Metastases. *Int. J. Radiat. Oncol. Biol. Phys.* **2017**, *99*, 22–30.
31. Yusuf, M.B.; Amsbaugh, M.J.; Burton, E.; Chesney, J.; Woo, S. Peri-SRS Administration of Immune Checkpoint Therapy for Melanoma Metastatic to the Brain: Investigating Efficacy and the Effects of Relative Treatment Timing on Lesion Response. *World Neurosurg.* **2017**, *100*, 632–640.
32. Chen, L.; Douglass, J.; Kleinberg, L.; Ye, X.; Marciscano, A.E.; Forde, P.M.; Brahmer, J.; Lipson, E.; Sharfman, W.; Hammers, H.; et al. Concurrent Immune Checkpoint Inhibitors and Stereotactic Radiosurgery for Brain Metastases in Non-Small Cell Lung Cancer, Melanoma, and Renal Cell Carcinoma. *Int J. Radiat. Oncol. Biol. Phys.* **2018**, *100*, 916–925.
33. Lanier, C.M.; Hughes, R.; Ahmed, T.; LeCompte, M.; Masters, A.H.; Petty, W.J.; Ruiz, J.; Triozzi, P.; Su, J.; O'Neill, S.; et al. Immunotherapy is associated with improved survival and decreased neurologic death after SRS for brain metastases from lung and melanoma primaries. *Neurooncol. Pract.* **2019**, *6*, 402–409.
34. Schapira, E.; Hubbeling, H.; Yeap, B.Y.; Mehan, W.A., Jr.; Shaw, A.T.; Oh, K.; Gainor, J.F.; Shih, H.A. Improved Overall Survival and Locoregional Disease Control With Concurrent PD-1 Pathway Inhibitors and Stereotactic Radiosurgery for Lung Cancer Patients With Brain Metastases. *Int. J. Radiat. Oncol. Biol. Phys.* **2018**, *101*, 624–629.
35. Shepard, M.J.; Xu, Z.; Donahue, J.; Eluvathingal Muttikkal, T.J.; Cordeiro, D.; Hansen, L.; Mohammed, N.; Gentzler, R.D.; Larner, J.; Fadul, C.E.; et al. Stereotactic radiosurgery with and without checkpoint inhibition for patients with metastatic non-small cell lung cancer to the brain: a matched cohort study. *J. Neurosurg.* **2019**, 1-8.
36. Koenig, J.L.; Shi, S.; Sborov, K.; Gensheimer, M.F.; Li, G.; Nagpal, S.; Chang, S.D.; Gibbs, I.C.; Soltys, S.G.; Pollom, E.L. Adverse Radiation Effect and Disease Control in Patients Undergoing Stereotactic Radiosurgery and Immune Checkpoint Inhibitor Therapy for Brain Metastases. *World Neurosurg.* **2019**, *126*, e1399–e1411.

37. Amaral, T.; Tampouri, I.; Eigentler, T.; Keim, U.; Klumpp, B.; Heinrich, V.; Zips, D.; Paulsen, F.; Gepfner-Tuma, I.; Skardelly, M.; et al. Immunotherapy plus surgery/radiosurgery is associated with favorable survival in patients with melanoma brain metastasis. *Immunotherapy* **2019**, *11*, 297–309.
38. Anderson, E.S.; Postow, M.A.; Wolchok, J.D.; Young, R.J.; Ballangrud, A.; Chan, T.A.; Yamada, Y.; Beal, K. Melanoma brain metastases treated with stereotactic radiosurgery and concurrent pembrolizumab display marked regression; efficacy and safety of combined treatment. *J. Immunother. Cancer* **2017**, *5*, e76.
39. Gabani, P.; Fischer-Valuck, B.W.; Johanns, T.M.; , H.; ez-Aya, L.F.; Keller, J.W.; Rich, K.M.; Kim, A.H.; Dunn, G.P.; Robinson, C.G.; et al. Stereotactic radiosurgery and immunotherapy in melanoma brain metastases: Patterns of care and treatment outcomes. *Radiother. Oncol.* **2018**, *128*, 266–273.
40. Galli, G.; Cavalieri, S.; Di Guardo, L.; Cimminiello, C.; Nichetti, F.; Corti, F.; Garcia, M.A.; Pappalardi, B.; Fallai, C.; De Braud, F.; et al. Combination of Immunotherapy and Brain Radiotherapy in Metastatic Melanoma: A Retrospective Analysis. *Oncol. Res. Treat.* **2019**, *42*, 182–188.
41. Kiess, A.P.; Wolchok, J.D.; Barker, C.A.; Postow, M.A.; Tabar, V.; Huse, J.T.; Chan, T.A.; Yamada, Y.; Beal, K. Stereotactic radiosurgery for melanoma brain metastases in patients receiving ipilimumab: safety profile and efficacy of combined treatment. *Int. J. Radiat. Oncol. Biol. Phys.* **2015**, *92*, 368–375.
42. Knisely, J.P.S.; Yu, J.B.; Flanigan, J.; Sznol, M.; Kluger, H.M.; Chiang, V.L.S. Radiosurgery for melanoma brain metastases in the ipilimumab era and the possibility of longer survival: Clinical article. *J. Neurosurg.* **2012**, *117*, 227–233.
43. Rahman, R.; Cortes, A.; Niemierko, A.; Oh, K.S.; Flaherty, K.T.; Lawrence, D.P.; Sullivan, R.J.; Shih, H.A. The impact of timing of immunotherapy with cranial irradiation in melanoma patients with brain metastases: intracranial progression, survival and toxicity. *J. Neurooncol.* **2018**, *138*, 299–306.
44. Rauschenberg, R.; Bruns, J.; Brutting, J.; Daubner, D.; Lohaus, F.; Zimmer, L.; Forschner, A.; Zips, D.; Hassel, J.C.; Berking, C.; et al. Impact of radiation, systemic therapy and treatment sequencing on survival of patients with melanoma brain metastases. *Eur. J. Cancer* **2019**, *110*, 11–20.
45. Schmidberger, H.; Rapp, M.; Ebersberger, A.; Hey-Koch, S.; Loquai, C.; Grabbe, S.; Mayer, A. Long-term survival of patients after ipilimumab and hypofractionated brain radiotherapy for brain metastases of malignant melanoma: sequence matters. *Strahlenther Onkol.* **2018**, *194*, 1144–1151.
46. Tazi, K.; Hathaway, A.; Chiuzan, C.; Shirai, K. Survival of melanoma patients with brain metastases treated with ipilimumab and stereotactic radiosurgery. *Cancer Med.* **2015**, *4*, 1–6.
47. Tetu, P.; Allayous, C.; Oriano, B.; Dalle, S.; Mortier, L.; Leccia, M.T.; Guillot, B.; Dalac, S.; Dutriaux, C.; Lacour, J.P.; et al. Impact of radiotherapy administered simultaneously with systemic treatment in patients with melanoma brain metastases within MelBase, a French multicentric prospective cohort. *Eur. J. Cancer* **2019**, *112*, 38–46.
48. Trommer-Nestler, M.; Marnitz, S.; Kocher, M.; Ruess, D.; Schlaak, M.; Theurich, S.; von Bergwelt-Baildon, M.; Morgenthaler, J.; Jablonska, K.; Celik, E.; et al. Robotic Stereotactic Radiosurgery in Melanoma Patients with Brain Metastases under Simultaneous Anti-PD-1 Treatment. *Int. J. Mol. Sci.* **2018**, *19*.
49. Ahmed, K.A.; Kim, S.; Arrington, J.; Naghavi, A.O.; Dilling, T.J.; Creelan, B.C.; Antonia, S.J.; Caudell, J.J.; Harrison, L.B.; Sahebjam, S.; et al. Outcomes targeting the PD-1/PD-L1 axis in conjunction with stereotactic radiation for patients with non-small cell lung cancer brain metastases. *J. Neurooncol.* **2017**, *133*, 331–338.
50. Singh, C.; Qian, J.M.; Yu, J.B.; Chiang, V.L. Local tumor response and survival outcomes after combined stereotactic radiosurgery and immunotherapy in non-small cell lung cancer with brain metastases. *J. Neurosurg.* **2019**, 1-6.
51. Kotecha, R.; Kim, J.M.; Miller, J.A.; Juloori, A.; Chao, S.T.; Murphy, E.S.; Peereboom, D.M.; Mohammadi, A.M.; Barnett, G.H.; Vogelbaum, M.A.; et al. The Impact of Sequencing PD-1/PD-L1 Inhibitors and Stereotactic Radiosurgery for Patients with Brain Metastasis. *Neuro-oncology* **2019**.
52. Shen, C.J.; Kummerlowe, M.N.; Redmond, K.J.; Rigamonti, D.; Lim, M.K.; Kleinberg, L.R. Stereotactic radiosurgery: Treatment of brain metastasis without interruption of systemic therapy. *Int. J. Radiat. Oncol. Biol. Phys.* **2016**, *95*, 735–742.

© 2020 by the authors. Licensee MDPI, Basel, Switzerland. This article is an open access article distributed under the terms and conditions of the Creative Commons Attribution (CC BY) license (http://creativecommons.org/licenses/by/4.0/).
